# Empirical Bayes Matrix Factorization

**Published:** 2021

**Authors:** Wei Wang, Matthew Stephens

**Affiliations:** Department of Statistics, University of Chicago, Chicago, IL, USA; Department of Statistics and Department of Human Genetics, University of Chicago, Chicago, IL, USA

**Keywords:** empirical Bayes, matrix factorization, normal means, sparse prior, unimodal prior, variational approximation

## Abstract

Matrix factorization methods, which include Factor analysis (FA) and Principal Components Analysis (PCA), are widely used for inferring and summarizing structure in multivariate data. Many such methods use a penalty or prior distribution to achieve sparse representations (“Sparse FA/PCA”), and a key question is how much sparsity to induce. Here we introduce a general Empirical Bayes approach to matrix factorization (EBMF), whose key feature is that it estimates the appropriate amount of sparsity by estimating prior distributions from the observed data. The approach is very flexible: it allows for a wide range of different prior families and allows that each component of the matrix factorization may exhibit a different amount of sparsity. The key to this flexibility is the use of a variational approximation, which we show effectively reduces fitting the EBMF model to solving a simpler problem, the so-called “normal means” problem. We demonstrate the benefits of EBMF with sparse priors through both numerical comparisons with competing methods and through analysis of data from the GTEx (Genotype Tissue Expression) project on genetic associations across 44 human tissues. In numerical comparisons EBMF often provides more accurate inferences than other methods. In the GTEx data, EBMF identifies interpretable structure that agrees with known relationships among human tissues. Software implementing our approach is available at https://github.com/stephenslab/flashr.

## 1. Introduction

Matrix factorization methods are widely used for inferring and summarizing structure in multivariate data. In brief, these methods represent an observed n×p data matrix Y as:

(1.1) $$Y=L^{T}F+E$$

where L is an K×n matrix, F is a K×p matrix, and E is an n×p matrix of residuals (whose entries we assume to be normally distributed, although the methods we develop can be generalized to other settings; see Section 6.4). Here we adopt the notation and terminology of factor analysis, and refer to L as the “loadings” and F as the “factors”.

The model (1.1) has many potential applications. One range of applications arise from “matrix completion” problems (e.g. Fithian et al., 2018): methods that estimate L and F in (1.1) from partially observed Y provide a natural and effective way to fill in the missing entries. Another wide range of applications come from the desire to *summarize and understand* the structure in a matrix *Y* : in (1.1) each row of Y is approximated by a linear combination of underlying “factors” (rows of), which—ideally—have some intuitive or scientific interpretation. For example, suppose $Y_{ij}$ represents the rating of a user i for a movie j. Each factor might represent a genre of movie (“comedy”, “drama”, “romance”, “horror” etc), and the ratings for a user i could be written as a linear combination of these factors, with the weights (loadings) representing how much individual i likes that genre. Or, suppose $Y_{ij}$ represents the expression of gene j in sample i. Each factor might represent a module of co-regulated genes, and the data for sample i could be written as a linear combination of these factors, with the loadings representing how active each module is in each sample. Many other examples could be given across many fields, including psychology (Ford et al., 1986), econometrics (Bai and Ng, 2008), natural language processing (Bouchard et al., 2015), population genetics (Engelhardt and Stephens, 2010), and functional genomics (Stein-O’Brien et al., 2018).

The simplest approaches to estimating L and/or F in (1.1) are based on maximum likelihood or least squares. For example, Principal Components Analysis (PCA)—or, more precisely, truncated Singular Value Decomposition (SVD)—can be interpreted as fitting (1.1) by least squares, assuming that columns of L are orthogonal and columns of F are orthonormal (Eckart and Young, 1936). And classical factor analysis (FA) corresponds to maximum likelihood estimation of L, assuming that the elements of F are independent standard normal and allowing different residual variances for each column of Y (Rubin and Thayer, 1982). While these simple methods remain widely used, in the last two decades researchers have focused considerable attention on obtaining more accurate and/or more interpretable estimates, either by imposing additional constraints (e.g. non-negativity; Lee and Seung, 1999) or by regularization using a penalty term (e.g. Jolliffe et al., 2003; Witten et al., 2009; Mazumder et al., 2010; Hastie et al., 2015; Fithian et al., 2018), or a prior distribution (e.g. Bishop, 1999; Attias, 1999; Ghahramani and Beal, 2000; West, 2003). In particular, many authors have noted the benefits of sparsity assumptions on L and/or F— particularly in applications where interpretability of the estimates is desired—and there now exists a wide range of methods that attempt to induce sparsity in these models (e.g. Sabatti and James, 2005; Zou et al., 2006; Pournara and Wernisch, 2007; Carvalho et al., 2008; Witten et al., 2009; Engelhardt and Stephens, 2010; Knowles and Ghahramani, 2011; Bhattacharya and Dunson, 2011; Mayrink et al., 2013; Yang et al., 2014; Gao et al., 2016; Hore et al., 2016; Ročková and George, 2016; Srivastava et al., 2017; Kaufmann and Schumacher, 2017; Frühwirth-Schnatter and Lopes, 2018; Zhao et al., 2018). Many of these methods induce sparsity in the loadings only, although some induce sparsity in both loadings and factors.

In any statistical problem involving sparsity, a key question is how strong the sparsity should be. In penalty-based methods this is controlled by the strength and form of the penalty, whereas in Bayesian methods it is controlled by the prior distributions. In this paper we take an Empirical Bayes approach to this problem, exploiting variational approximation methods (Blei et al., 2017) to obtain simple algorithms that jointly estimate the prior distributions for both loadings and factors, as well as the loadings and factors themselves.

Both EB and variational methods have been previously used for this problem (Bishop, 1999; Lim and Teh, 2007; Raiko et al., 2007; Stegle et al., 2010). However, most of this previous work has used simple normal prior distributions that do not induce sparsity. Variational methods that use sparsity-inducing priors include Girolami (2001), which uses a Laplace prior on the factors (no prior on the loadings which are treated as free parameters), Hochreiter et al. (2010) which extends this to Laplace priors on both factors and loadings, with fixed values of the Laplace prior parameters; Titsias and Lázaro-Gredilla (2011), which uses a sparse “spike-and-slab” (point normal) prior on the loadings (with the same prior on all K loadings) and a normal prior on the factors; and Hore et al. (2016) which uses a spike-and-slab prior on one mode in a tensor decomposition. (While this work was in review further examples appeared, including Argelaguet et al. 2018, which uses normal priors on the loadings and point-normal on the factors.)

Our primary contribution here is to develop and implement a more general EB approach to matrix factorization (EBMF). This general approach allows for a wide range of potential sparsity-inducing prior distributions on both the loadings and the factors within a single algorithmic framework. We accomplish this by showing that, when using variational methods, fitting EBMF with *any* prior family can be reduced to repeatedly solving a much simpler problem—the “empirical Bayes normal means” (EBNM) problem—with the same prior family. This feature makes it easy to implement methods for any desired prior family—one simply has to implement a method to solve the corresponding normal means problem, and then plug this into our algorithm. This approach can work for both parametric families (e.g. normal, point-normal, laplace, point-laplace) and non-parametric families, including the “adaptive shrinkage” priors (unimodal and scale mixtures of normals) from Stephens (2017). It is also possible to accommodate non-negative constraints on either L and/or F by using non-negative prior families. Even simple versions of our approach—e.g. using point-normal priors on both factors and loadings— provide more generality than most existing EBMF approaches and software.

A second contribution of our work is to highlight similarities and differences between EBMF and penalty-based methods for regularizing L and/or F. Indeed, our algorithm for fitting EBMF has the same structure as commonly-used algorithms for penalty-based methods, with the prior distribution playing a role analogous to the penalty (see Remark 3 later). While the general correspondence between estimates from penalized methods and Bayesian posterior modes (MAP estimates) is well known, the connection here is different, because the EBMF approach is estimating a posterior mean, not a mode (indeed, with sparse priors the MAP estimates of L and F are not useful because they are trivially 0). A key difference between the EBMF approach and penalty-based methods is that the EBMF prior is estimated by solving an optimization problem, whereas in penalty-based methods the strength of the penalty is usually chosen by cross-validation. This difference makes it much easier for EBMF to allow for different levels of sparsity in every factor and every loading: in EBMF one simply uses a different prior for every factor and loading, whereas tuning a separate parameter for every factor and loading by CV becomes very cumbersome.

The final contribution is that we provide an R software package, *flash* (Factors and Loadings by Adaptive SHrinkage), implementing our flexible EBMF framework. We demonstrate the utility of these methods through both numerical comparisons with competing methods and through a scientific application: analysis of data from the GTEx (Genotype Tissue Expression) project on genetic associations across 44 human tissues. In numerical comparisons *flash* often provides more accurate inferences than other methods, while remaining computationally tractable for moderate-sized matrices (millions of entries). In the GTEx data, *flash* highlight both effects that are shared across many tissues (“dense” factors) and effects that are specific to a small number of tissues (“sparse” factors). These sparse factors often highlight similarities between tissues that are known to be biologically related, providing external support for the reliability of the results.

## 2. A General Empirical Bayes Matrix Factorization Model

We define the K-factor Empirical Bayes Matrix Factorization (EBMF) model as follows:

(2.1) $$Y=\sum_{k=1}^{K}\;\;l_{k}f_{k}^{T}+E$$

(2.2) $$l_{k1},\ldots ,l_{kn}\sim^{iid}g_{l_{k}},\;g_{l_{k}}\in {𝒢}_{l}$$

(2.3) $${f_{k1},\ldots ,f_{kp}\sim }^{iid}g_{f_{k}},\;g_{f_{k}}\in {𝒢}_{f}$$

(2.4) $$E_{ij}\;\sim N\left(0,1/\tau_{ij}\right)\text{ with }\tau ≔\left(\tau_{ij}\right)\in 𝒯.$$

Here Y is the n×p observed data matrix, $l_{k}$ is an n-vector (the kth set of “loadings”), $f_{k}$ is a p-vector (the kth “factor”), ${𝒢}_{l}$ and ${𝒢}_{f}$ are pre-specified (possibly non-parametric) families of distributions, $g_{l_{k}}$ and $g_{f_{k}}$ are unknown “prior” distributions that are to be estimated, E is an n×p matrix of independent error terms, and τ is an unknown n×p matrix of precisions $\left(\tau_{ij}\right)$ which is assumed to lie in some space 𝒯. (This allows structure to be imposed on τ, such as constant precision, $\tau_{ij}=\tau$, or column-specific precisions, $\tau_{ij}=\tau_{j}$, for example.) Our methods allow that some elements of Y may be “missing”, and can estimate the missing values (Section 4.1).

The term “Empirical Bayes” in EBMF means we fit (2.1)-(2.4) by obtaining *point estimates* for the priors $g_{l_{k}},g_{f_{k}},(k=1,\ldots ,K)$ and approximate the posterior distributions for the parameters $l_{k},f_{k}$ given those point estimates. This contrasts with a “fully Bayes” approach that, instead of obtaining point estimates for $g_{l_{k}},g_{f_{k}}$, would integrate over uncertainty in the estimates. This would involve specifying prior distributions for $g_{l_{k}},g_{f_{k}}$ as well as (perhaps substantial) additional computation. The EB approach has the advantage of simplicity - both conceptually and computationally—while enjoying many of the benefits of a fully Bayes approach. In particular it allows for sharing of information across elements of each loading/factor. For example, if the data suggest that a particular factor, $f_{k}$, is sparse, then this will be reflected in a sparse estimate of $g_{f_{k}}$, and subsequently strong shrinkage of the smaller elements of $f_{k1},\ldots ,f_{kp}$ towards 0. Conversely, when the data suggest a non-sparse factor then the prior will be dense and the shrinkage less strong. By allowing different prior distributions for each factor and each loading, the model has the flexibility to adapt to any combination of sparse and dense loadings and factors. However, to fully capitalize on this flexibility one needs suitably flexible prior families ${𝒢}_{l}$ and ${𝒢}_{f}$ capable of capturing both sparse and dense factors. A key feature of our work is it allows for very flexible prior families, including non-parametric families.

Some specific choices of the distributional families ${𝒢}_{l}$ and ${𝒢}_{f}$ correspond to models used in previous work. In particular, many previous papers have studied the case with normal priors, where ${𝒢}_{l}$ and ${𝒢}_{f}$ are both the family of zero-mean normal distributions (e.g. Bishop, 1999; Lim and Teh, 2007; Raiko et al., 2007; Nakajima and Sugiyama, 2011). This family is particularly simple, having a single hyper-parameter, the prior variance, to estimate for each factor. However, it does not induce sparsity on either L or F; indeed, when the matrix Y is fully observed, the estimates of L and F under a normal prior (when using a fully factored variational approximation) are simply scalings of the singular vectors from an SVD of Y (Nakajima and Sugiyama, 2011; Nakajima et al., 2013). Our work here extends these previous approaches to a much wider range of prior families that do induce sparsity on L and/or F.

We note that the EBMF model (2.1)-(2.4) differs in an important way from the sparse factor analysis (SFA) methods in Engelhardt and Stephens (2010), which use a type of Automatic Relevance Determination prior (e.g. Tipping, 2001; Wipf and Nagarajan, 2008) to induce sparsity on the loadings matrix. In particular, SFA estimates a separate hyperparameter for every element of the loadings matrix, with no sharing of information across elements of the same loading. In contrast, EBMF estimates a single *shared* prior distribution for elements of each loading, which, as noted above, allows for sharing of information across elements of each loading/factor.

## 3. Fitting the EBMF Model

To simplify exposition we begin with the case K=1 (“rank 1”); see Section 3.6 for the extension to general K. To simplify notation we assume the families ${𝒢}_{l},{𝒢}_{f}$ are the same, so we can write ${𝒢}_{l}={𝒢}_{f}=𝒢$. To further lighten notation in the case K=1 we use $g_{l},g_{f},l,f$ instead of $g_{l_{1}},g_{f_{1}},l_{1},f_{1}$.

Fitting the EBMF model involves estimating all of $g_{l},g_{f},l,f,\tau$. A standard EB approach would be to do this in two steps:

- Estimate $g_{l},g_{f}$ and τ, by maximizing the likelihood:
(3.1) $$L\left(g_{l},g_{f},\tau \right)≔\iint \;p(Y|l,f,\tau )g_{l}\left(dl_{1}\right)\ldots g_{l}\left(dl_{n}\right)g_{f}\left(df_{1}\right)\ldots g_{f}\left(df_{p}\right)$$
  over $g_{l},g_{f}\in 𝒢,\tau \in 𝒯$. (This optimum will typically not be unique because of identifiability issues; see Section 3.8.)
- Estimate l and f using their posterior distribution: $p\left(l,f|Y,{\overset{ˆ}{g}}_{l},{\overset{ˆ}{g}}_{f},\overset{ˆ}{\tau }\right)$.

However, both these two steps are difficult, even for very simple choices of 𝒢. Instead, following previous work (see Introduction for citations) we use variational approximations to approximate this approach. Although variational approximations are known to typically under-estimate uncertainty in posterior distributions, our focus here is on obtaining useful point estimates for l,f; results shown later demonstrate that the variational approximation can perform well in this task.

### 3.1. The Variational Approximation

The variational approach—see Blei et al. (2017) for review—begins by writing the log of the likelihood (3.1) as:

(3.2) $$l\left(g_{l},g_{f},\tau \right)\;≔\text{log}L\left(g_{l},g_{f},\tau \right)$$

(3.3) $$\begin{array}{rr} & \;=F\left(q,g_{l},g_{f},\tau \right)+D_{KL}(q∥p) \end{array}$$

where

(3.4) $$F\left(q,g_{l},g_{f},\tau \right)=\int \;q(l,f)\text{log}\frac{p\left(Y,l,f|g_{l},g_{f},\tau \right)}{q(l,f)}dldf,$$

and

(3.5) $$D_{KL}(q||p)=-\int \;q(l,f)\text{log}\frac{p\left(l,f|Y,g_{l},g_{f},\tau \right)}{q(l,f)}dldf$$

is the Kullback–Leibler divergence from q to p. This identity holds for any distribution q(l,f). Because $D_{KL}$ is non-negative, it follows that $F\left(q,g_{l},g_{f},\tau \right)$ is a lower bound for the log likelihood:

(3.6) $$l\left(g_{l},g_{f},\tau \right)\geq F\left(q,g_{l},g_{f},\tau \right)$$

with equality when $q(l,f)=p\left(l,f|Y,g_{l},g_{f},\tau \right)$.

In other words,

(3.7) $$l\left(g_{l},g_{f},\tau \right)=\underset{q}{\text{max}}\;F\left(q,g_{l},g_{f},\tau \right),$$

where the maximization is over all possible distributions q(l,f). Maximizing $l\left(g_{l},g_{f},\tau \right)$ can thus be viewed as maximizing F over $q,g_{l},g_{f},\tau$. However, as noted above, this maximization is difficult. The variational approach simplifies the problem by maximizing F but restricting the family of distributions for q. Specifically, the most common variational approach — and the one we consider here—restricts q to the family 𝒬 of distributions that “fully-factorize”:

(3.8) $$𝒬=\left\{q:q(l,f)=\prod_{i=1}^{n}\;\;q_{l,i}\left(l_{i}\right)\prod_{j=1}^{p}\;\;q_{f,j}\left(f_{j}\right)\right\}.$$

The variational approach seeks to optimize F over $q,g_{l},g_{f},\tau$ with the constraint q∈Q. For q∈Q we can write $q(l,f)=q_{l}(l)q_{f}(f)$ where $q_{l}(l)=\prod_{i=1}^{n}\;q_{l,i}\left(l_{i}\right)$ and $q_{f}(f)=$ $\prod_{j=1}^{p}\;q_{f,j}\left(f_{j}\right)$, and we can consider the problem as maximizing $F\left(q_{l},q_{f},g_{l},g_{f},\tau \right)$.

### 3.2. Alternating Optimization

We optimize $F\left(q_{l},q_{f},g_{l},g_{f},\tau \right)$ by alternating between optimizing over variables related to *l* $\left[\left(q_{l},g_{l}\right)\right]$, over variables related to $f\left[\left(q_{f},g_{f}\right)\right]$, and over τ. Each of these steps is guaranteed to increase (or, more precisely, not decrease) F, and convergence can be assessed by (for example) stopping when these optimization steps yield a very small increase in F. Note that F may be multi-modal, and there is no guarantee that the algorithm will converge to a global optimum. The approach is summarized in Algorithm 1


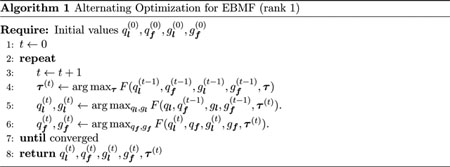


The key steps in Algorithm 1 are the maximizations in Steps 4–6.

Step 4, the update of τ, involves computing the expected squared residuals:

(3.9) $${\bar{R^{2}}}_{ij}≔{\text{E}}_{q_{l},q_{f}}\left[{\left(Y_{ij}-l_{i}f_{j}\right)}^{2}\right]$$

(3.10) $$\begin{array}{rr} & \;={\left[Y_{ij}-{\text{E}}_{q_{l}}\left(l_{i}\right){\text{E}}_{q_{f}}\left(f_{j}\right)\right]}^{2}-{\text{E}}_{q_{l}}{\left(l_{i}\right)}^{2}{\text{E}}_{q_{f}}{\left(f_{j}\right)}^{2}+{\text{E}}_{q_{l}}\left(l_{i}^{2}\right){\text{E}}_{q_{f}}\left(f_{j}^{2}\right). \end{array}$$

This is straightforward provided the first and second moments of $q_{l}$ and $q_{f}$ are available (see Appendix A.1 for details).

Steps 5 and 6 are essentially identical except for switching the role of l and f. One of our key results is that each of these steps can be achieved by solving a simpler problem—the Empirical Bayes normal means (EBNM) problem. The next subsection (3.3) describes the EBNM problem, and the following subsection (3.4) details how this can be used to solve Steps 5 and 6.

### 3.3. The EBNM Problem

Suppose we have observations $x=\left(x_{1},\ldots ,x_{n}\right)$ of underlying quantities $\theta =\left(\theta_{1},\ldots ,\theta_{n}\right)$, with independent Gaussian errors with known standard deviations $s=\left(s_{1},\ldots ,s_{n}\right)$. Suppose further that the elements of θ are assumed i.i.d. from some distribution, g∈𝒢. That is,

(3.11) $$x|\theta \sim N_{n}\left(\theta ,\text{diag}\left(s_{1}^{2},\ldots ,s_{n}^{2}\right)\right)$$

(3.12) $$\begin{array}{rr} \theta_{1},\ldots ,\theta_{n} & \;\sim^{\text{iid }}g,\;g\in 𝒢 \end{array}$$

where $N_{n}(\mu ,\Sigma )$ denotes the n-dimensional normal distribution with mean μ and covariance matrix Σ.

By solving the EBNM problem we mean fitting the model (3.11)-(3.12) by the following two-step procedure:

1. Estimate g by maximum (marginal) likelihood:
(3.13) $$\overset{ˆ}{g}=\text{arg}\underset{g\in 𝒢}{\text{max}}\;\prod_{j}\;\int \;p\left(x_{j}|\theta_{j},s_{j}\right)g\left(d\theta_{j}\right).$$
2. Compute the posterior distribution for θ given $\overset{ˆ}{g}$,
(3.14) $$p(\theta |x,s,\overset{ˆ}{g})\propto \prod_{j}\;\overset{ˆ}{g}\left(\theta_{j}\right)p\left(x_{j}|\theta_{j},s_{j}\right).$$

Later in this paper we will have need for the posterior first and second moments, so we define them here for convenience:

(3.15) $${\overset{‾}{\theta }}_{j}\;≔E\left(\theta_{j}|x,s,\overset{ˆ}{g}\right)$$

(3.16) $${\bar{\theta^{2}}}_{j}\;≔E\left(\theta_{j}^{2}|x,s,\overset{ˆ}{g}\right).$$

Formally, this procedure defines a mapping (which depends on the family 𝒢) from the known quantities (x,s), to $\left(\overset{ˆ}{g},p\right)$, where $\overset{ˆ}{g},p$ are given in (3.13) and (3.14). We use EBNM to denote this mapping:

(3.17) $$\text{EBNM}(x,s)=(\overset{ˆ}{g},p)$$

**Remark 1** Solving the EBNM problem is central to all our algorithms, so it is worth some study. A key point is that the EBNM problem provides an attractive and flexible way to induce shrinkage and/or sparsity in estimates of θ. For example, if θ is truly sparse, with many elements at or near 0, then the estimate $\overset{ˆ}{g}$ will typically have considerable mass near 0, and the posterior means (3.15) will be “shrunk” strongly toward 0 compared with the original observations. In this sense solving the EBNM problem can be thought of as a model-based analogue of thresholding-based methods, with the advantage that by estimating g from the data the EBNM approach automatically adapts to provide an appropriate level of shrinkage. These ideas have been used in wavelet denoising (Clyde and George, 2000; Johnstone et al., 2004; Johnstone and Silverman, 2005a; Xing et al., 2016), and false discovery rate estimation (Thomas et al., 1985; Stephens, 2017) for example. Here we apply them to matrix factorization problems.

### 3.4. Connecting the EBMF and EBNM Problems

The EBNM problem is well studied, and can be solved reasonably easily for many choices of 𝒢 (e.g. Johnstone and Silverman, 2005b; Koenker and Mizera, 2014a; Stephens, 2017). In Section 4 we give specific examples; for now our main point is that *if* one can solve the EBNM problem for a particular choice of 𝒢 then it can be used to implement Steps 5 and 6 in Algorithm 1 for the corresponding EBMF problem. The following Proposition formalizes this for Step 5 of Algorithm 1; a similar proposition holds for Step 6 (see also Appendix A).

**Proposition 2** Step 5 in Algorithm 1 is solved by solving an EBNM problem. Specifically

(3.18) $$\text{arg}\underset{q_{l},g_{l}}{\text{max}}\;F\left(q_{l},q_{f},g_{l},g_{f},\tau \right)=\text{EBNM}\left(\overset{ˆ}{l}\left(Y,\overset{-}{f},\overset{-}{f^{2}},\tau \right),s_{l}\left(\overset{-}{f^{2}},\tau \right)\right)$$

where the functions $\overset{ˆ}{l}:R^{n\times p}\times R^{p}\times R^{p}\times R^{n\times p}\to R^{n}$ and $s_{l}:R^{p}\times R^{n\times p}\to R^{n}$ are given by

(3.19) $$\overset{ˆ}{l}(Y,v,w,\tau {)}_{i}\;≔\frac{\sum_{j}\;\;\tau_{ij}Y_{ij}v_{j}}{\sum_{j}\;\;\tau_{ij}w_{j}},$$

(3.20) $$s_{l}(w,\tau {)}_{i}\;≔{\left(\sum_{j}\;\;\tau_{ij}w_{j}\right)}^{-0.5},$$

and $\overset{-}{f},\overset{-}{f^{2}}\in R^{p}$ denote the vectors whose elements are the first and second moments of f under $q_{f}$:

(3.21) $$\overset{-}{f}\;≔\left(E_{q_{f}}\left(f_{j}\right)\right)$$

(3.22) $$\overset{-}{f^{2}}\;≔\left(E_{q_{f}}\left(f_{j}^{2}\right)\right).$$

**Proof** See Appendix A. ■

For intuition into where the EBNM in Proposition 2 comes from, consider estimating $l,g_{l}$ in (2.1) with f and τ known. The model then becomes n independent regressions of the rows of Y on f, and the maximum likelihood estimate for l has elements:

(3.23) $${\overset{ˆ}{l}}_{i}=\frac{\sum_{j}\;\;\tau_{ij}Y_{ij}f_{j}}{\sum_{j}\;\;\tau_{ij}f_{j}^{2}},$$

with standard errors

(3.24) $$s_{i}={\left(\sum_{j}\;\;\tau_{ij}f_{j}^{2}\right)}^{-0.5}.$$

Further, it is easy to show that

(3.25) $${\overset{ˆ}{l}}_{i}\sim N\left(l_{i},s_{i}^{2}\right).$$

Combining (3.25) with the prior

(3.26) $$l_{1},\ldots ,l_{n}\sim^{iid}g_{l},\;g_{l}\in 𝒢$$

yields an EBNM problem.

The EBNM in Proposition 2 is the same as the EBNM (3.25)-(3.26), but with the terms $f_{j}$ and $f_{j}^{2}$ replaced with their expectations under $q_{f}$. Thus, the update for $\left(q_{l},g_{l}\right)$ in Algorithm 1, with $\left(q_{f},g_{f},\tau \right)$ fixed, is closely connected to solving the EBMF problem for “known f,τ “.

### 3.5. Streamlined Implementation Using First and Second Moments

Although Algorithm 1, as written, optimizes over $\left(q_{l},q_{f},g_{l},g_{f}\right)$, in practice each step requires only the first and second moments of the distributions $q_{l}$ and $q_{f}$. For example, the EBNM problem in Proposition 1 involves $\overset{-}{f}$ and $\overset{-}{f^{2}}$ and not $g_{f}$. Consequently, we can simplify implementation by keeping track of only those moments. In particular, when solving the normal means problem, EBNM⁡(x,s) in (3.17), we need only return the posterior first and second moments (3.15) and (3.16). This results in a streamlined and intuitive implementation, summarized in Algorithm 2.


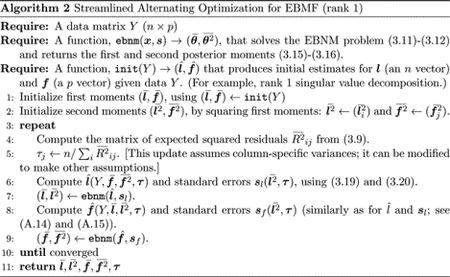


**Remark 3** Algorithm 2 has a very intuitive form: it has the flavor of an alternating least squares algorithm, which alternates between estimating l given f (Step 6) and f given l (Step 8), but with the addition of the ebnm step (Steps 7 and 9), which can be thought of as regularizing or shrinking the estimates: see Remark 1. This viewpoint highlights connections with related algorithms. For example, the (rank 1 version of the) SSVD algorithm from Yang et al. (2014) has a similar form, but uses a thresholding function in place of the ebnm function to induce shrinkage and/or sparsity.

### 3.6. The K-factor EBMF Model

It is straightforward to extend the variational approach to fit the general K factor model (2.1)-(2.4). In brief, we introduce variational distributions $\left(q_{l_{k}},q_{f_{k}}\right)$ for k=1,…,K, and then optimize the objective function $F\left(q_{l_{1}},g_{l_{1}},q_{f_{1}},g_{f_{1}};\ldots ;q_{l_{K}},g_{l_{K}},q_{f_{K}},g_{f_{K}};\tau \right)$. Similar to the rank-1 model, this optimization can be done by iteratively updating parameters relating to a single loading or factor, keeping other parameters fixed. And again we simplify implementation by keeping track of only the first and second moments of the distributions $q_{l_{k}}$ and $q_{f_{k}}$, which we denote ${\overset{-}{l}}_{k},{\overset{-}{l}}_{k}^{2},{\overset{-}{f}}_{k},{\overset{-}{f}}_{k}^{2}$. The updates to ${\overset{-}{l}}_{k},{\overset{-}{l}}_{k}{\;}^{\text{(and }}{\overset{-}{f}}_{k},{\overset{-}{f}}_{k}^{2}$) are essentially identical to those for fitting the rank 1 model above, but with $Y_{ij}$ replaced with the residuals obtained by removing the estimated effects of the other k-1 factors:

(3.27) $$R_{ij}^{k}≔Y_{ij}-\sum_{k^{\prime }\neq k}\;{\overset{-}{l}}_{k^{\prime }i}{\overset{-}{f}}_{k^{\prime }j}.$$

Based on this approach we have implemented two algorithms for fitting the K-factor model. First, a simple “greedy” algorithm, which starts by fitting the rank 1 model, and then adds factors k=2,…,K, one at a time, optimizing over the new factor parameters before moving on to the next factor. Second, a “backfitting” algorithm (Breiman and Friedman, 1985), which iteratively refines the estimates for each factor given the estimates for the other factors. Both algorithms are detailed in Appendix A.

### 3.7. Selecting K

An interesting feature of EB approaches to matrix factorization, noted by Bishop (1999), is that they automatically select the number of factors K. This is because the maximum likelihood solution to $g_{l_{k}},g_{f_{k}}$ is sometimes a point mass on 0 (provided 𝒢 includes this distribution). Furthermore, the same is true of the solution to the variational approximation (see also Bishop, 1999; Stegle et al., 2012). This means that if K is set sufficiently large then some loading/factor combinations will be optimized to be exactly 0. (Or, in the greedy approach, which adds one factor at a time, the algorithm will eventually add a factor that is exactly 0, at which point it terminates.)

Here we note that the variational approximation may be expected to result in conservative estimation (i.e. underestimation) of K compared with the (intractable) use of maximum likelihood to estimate $g_{l},g_{f}$. We base our argument on the simplest case: comparing K=1 vs K=0. Let $\delta_{0}$ denote the degenerate distribution with all its mass at 0. Note that the rank-1 factor model (2.1), with $g_{l}=\delta_{0}$ (or $g_{f}=\delta_{0}$) is essentially a “rank-0” model. Now note that the variational lower bound, F, is exactly equal to the log-likelihood when $g_{l}=\delta_{0}$ (or $g_{f}=\delta_{0}$). This is because if the prior is a point mass at 0 then the posterior is also a point mass, which trivially factorizes as a product of point masses, and so the variational family 𝒬 includes the true posterior in this case. Since F is a lower bound to the log-likelihood we have the following simple lemma:

**Lemma 4** If $F\left(\overset{ˆ}{q},{\overset{ˆ}{g}}_{l},{\overset{ˆ}{g}}_{f},\overset{ˆ}{\tau }\right)>F\left(\delta_{0},\delta_{0},\delta_{0},{\overset{ˆ}{\tau }}_{0}\right)$ then $l\left({\overset{ˆ}{g}}_{l},{\overset{ˆ}{g}}_{f},\overset{ˆ}{\tau }\right)>l\left(\delta_{0},\delta_{0},{\overset{ˆ}{\tau }}_{0}\right)$.

Proof

(3.28) $$l\left({\overset{ˆ}{g}}_{l},{\overset{ˆ}{g}}_{f},\overset{ˆ}{\tau }\right)\geq F\left(\overset{ˆ}{q},{\overset{ˆ}{g}}_{l},{\overset{ˆ}{g}}_{f},\overset{ˆ}{\tau }\right)>F\left(\delta_{0},\delta_{0},\delta_{0},{\overset{ˆ}{\tau }}_{0}\right)=l\left(\delta_{0},\delta_{0},{\overset{ˆ}{\tau }}_{0}\right)$$

■

Thus, if the variational approximation F favors ${\overset{ˆ}{g}}_{l},{\overset{ˆ}{g}}_{f},\overset{ˆ}{\tau }$ over the rank 0 model, then it is guaranteed that the likelihood would also favor ${\overset{ˆ}{g}}_{l},{\overset{ˆ}{g}}_{f},\overset{ˆ}{\tau }$ over the rank 0 model. In other words, compared with the likelihood, the variational approximation is conservative in terms of preferring the rank 1 model to the rank 0 model. This conservatism is a double-edged sword. On the one hand it means that if the variational approximation finds structure it should be taken seriously. On the other hand it means that the variational approximation could miss subtle structure.

In practice Algorithm 2 can converge to a local optimum of F that is not as high as the trivial (rank 0) solution, $F\left(\delta_{0},\delta_{0},\delta_{0},{\overset{ˆ}{\tau }}_{0}\right)$. We can add a check for this at the end of Algorithm 2, and set ${\overset{ˆ}{g}}_{l}={\overset{ˆ}{g}}_{f}=\delta_{0}$ and $\overset{ˆ}{\tau }={\overset{ˆ}{\tau }}_{0}$ when this occurs.

### 3.8. Identifiability

In EBMF each loading and factor is identifiable, at best, only up to a multiplicative constant (provided 𝒢 is a scale family). Specifically, scaling the prior distributions $g_{f_{k}}$ and $g_{l_{k}}$ by $c_{k}$ and $1/c_{k}$ respectively results in the same marginal likelihood, and also results in a corresponding scaling of the posterior distribution on the factors $f_{k}$ and loadings $l_{k}$ (e.g. it scales the posterior first moments by $c_{k},1/c_{k}$ and the second moments by $\left.c_{k}^{2},1/c_{k}^{2}\right)$. However, this non-identifiability is not generally a problem, and if necessary it could be dealt with by re-scaling factor estimates to have norm 1.

## 4. Software Implementation: *flash*

We have implemented Algorithms 2, 4 and 5 in an R package, flash (“factors and loadings via adaptive shrinkage”). These algorithms can fit the EBMF model for any choice of distributional family ${𝒢}_{l},{𝒢}_{f}$: the user must simply provide a function to solve the EBNM problem for these prior families.

One source of functions for solving the EBNM problem is the “adaptive shrinkage” (ashr) package, which implements methods from Stephens (2017). These methods solve the EBNM problem for several flexible choices of 𝒢, including:

- 𝒢=𝒮N, the set of all scale mixtures of zero-centered normals;
- 𝒢=𝒮𝒰, the set of all symmetric unimodal distributions, with mode at 0;
- 𝒢=𝒰, the set of all unimodal distributions, with mode at 0;
- $𝒢={𝒰}_{+}$, the set of all non-negative unimodal distributions, with mode at 0.

These methods are computationally stable and efficient, being based on convex optimization methods (Koenker and Mizera, 2014b) and analytic Bayesian posterior computations.

We have also implemented functions to solve the EBNM problem for additional choices of 𝒢 in the package ebnm (https://github.com/stephenslab/ebnm). These include 𝒢 being the “point-normal” family:

- 𝒢=𝒫N, the set of all distributions that are a mixture of a point mass at zero and a normal with mean 0.

This choice is less flexible than those in ashr, and involves non-convex optimizations, but can be faster.

Although in this paper we focus our examples on sparsity-inducing priors with ${𝒢}_{l}={𝒢}_{f}=𝒢$ we note that our software makes it easy to experiment with different choices, some of which represent novel methodologies. For example, setting ${𝒢}_{l}={𝒰}_{+}$ and ${𝒢}_{f}=𝒮N$ yields an EB version of semi-non-negative matrix factorization (Ding et al., 2008), and we are aware of no existing EB implementations for this problem. Exploring the relative merits of these many possible options in different types of application will be an interesting direction for future work.

### 4.1. Missing Data

If some elements of Y are missing, then this is easily dealt with. For example, the sums over j in (3.19) and (3.20) are simply computed using only the j for which $Y_{ij}$ is not missing. This corresponds to an assumption that the missing elements of Y are “missing at random” (Rubin, 1976). In practice we implement this by setting $\tau_{ij}=0$ whenever $Y_{ij}$ is missing (and filling in the missing entries of Y to an arbitrary number). This allows the implementation to exploit standard fast matrix multiplication routines, which cannot handle missing data. If many data points are missing then it may be helpful to exploit sparse matrix routines.

### 4.2. Initialization

Both Algorithms 2 and 4 require a rank 1 initialization procedure, init. Here, we use the softImpute function from the package softImpute (Mazumder et al., 2010), with penalty parameter λ=0, which essentially performs SVD when Y is completely observed, but can also deal with missing values in Y.

The backfitting algorithm (Algorithm 5) also requires initialization. One option is to use the greedy algorithm to initialize, which we call “greedy+backfitting”.

## 5. Numerical Comparisons

We now compare our methods with several competing approaches. To keep these comparisons manageable in scope we focus attention on methods that aim to capture possible sparsity in L and/or F. For EBMF we present results for two different shrinkage-oriented prior families, 𝒢: the scale mixture of normals (𝒢=𝒮N), and the point-normal family (𝒢=𝒫N). We denote these *flash* and *flash*_pn respectively when we need to distinguish. In addition we consider Sparse Factor Analysis (SFA) (Engelhardt and Stephens, 2010), SFAmix (Gao et al., 2013), Nonparametric Bayesian Sparse Factor Analysis (NBSFA) (Knowles and Ghahramani, 2011), Penalized Matrix Decomposition (Witten et al., 2009) (PMD, implemented in the R package PMA), and Sparse SVD (Yang et al., 2014) (SSVD, implemented in R package ssvd). Although the methods we compare against involve only a small fraction of the very large number of methods for this problem, the methods were chosen to represent a wide range of different approaches to inducing sparsity: SFA, SFAmix and NBSFA are three Bayesian approaches with quite different approaches to prior specification; PMD is based on a penalized likelihood with $L_{1}$ penalty on factors and/or loadings; and SSVD is based on iterative thresholding of singular vectors. We also compare with softImpute (Mazumder et al., 2010), which does not explicitly model sparsity in L and F, but fits a regularized low-rank matrix using a nuclear-norm penalty. Finally, for reference we also use standard (truncated) SVD.

All of the Bayesian methods (*flash*, SFA, SFAmix and NBSFA) are “self-tuning”, at least to some extent, and we applied them here with default values. According to Yang et al. (2014) SSVD is robust to choice of tuning parameters, so we also ran SSVD with its default values, using the robust option (method=“method”). The softImpute method has a single tuning parameter (λ, which controls the nuclear norm penalty), and we chose this penalty by orthogonal cross-validation (OCV; Appendix B). The PMD method can use two tuning parameters (one for l and one for *f*) to allow different sparsity levels in l vs f. However, since tuning two parameters can be inconvenient it also has the option to use a single parameter for both l and f. We used OCV to tune parameters in both cases, referring to the methods as PMD.cv2 (2 tuning parameters) and PMD.cv1 (1 tuning parameter).

### 5.1. Simple Simulations

#### 5.1.1. A Single Factor Example

We simulated data with n=200,p=300 under the single-factor model (2.1) with sparse loadings, and a non-sparse factor:

(5.1) $$l_{i}\sim \pi_{0}\delta_{0}+\left(1-\pi_{0}\right)\overset{5}{\underset{m=1}{\sum }}\frac{1}{5}N\left(0,\sigma_{m}^{2}\right)$$

(5.2) $$f_{j}\sim N(0,1)$$

where $\delta_{0}$ denotes a point mass on 0, and $\left(\sigma_{1}^{2},\ldots ,\sigma_{5}^{2}\right)≔(0.25,0.5,1,2,4)$. We simulated using three different levels of sparsity on the loadings, using $\pi_{0}=0.9,0.3,0$. (We set the noise precision τ=1,1/16,1/25 in these three cases to make each problem not too easy and not too hard.)

We applied all methods to this rank-1 problem, specifying the true value K=1. (The NBSFA software does not provide the option to fix K, so is omitted here.) We compare methods in their accuracy in estimating the true low-rank structure $\left(B≔lf^{T}\right)$ using relative root mean squared error:

(5.3) $$\text{RRMSE}(\overset{ˆ}{B},B)≔\sqrt{\frac{\sum_{i,j}\;\;{\left({\overset{ˆ}{B}}_{ij}-B_{ij}\right)}^{2}}{\sum_{i,j}\;\;B_{ij}^{2}}}.$$

**Figure 1:**
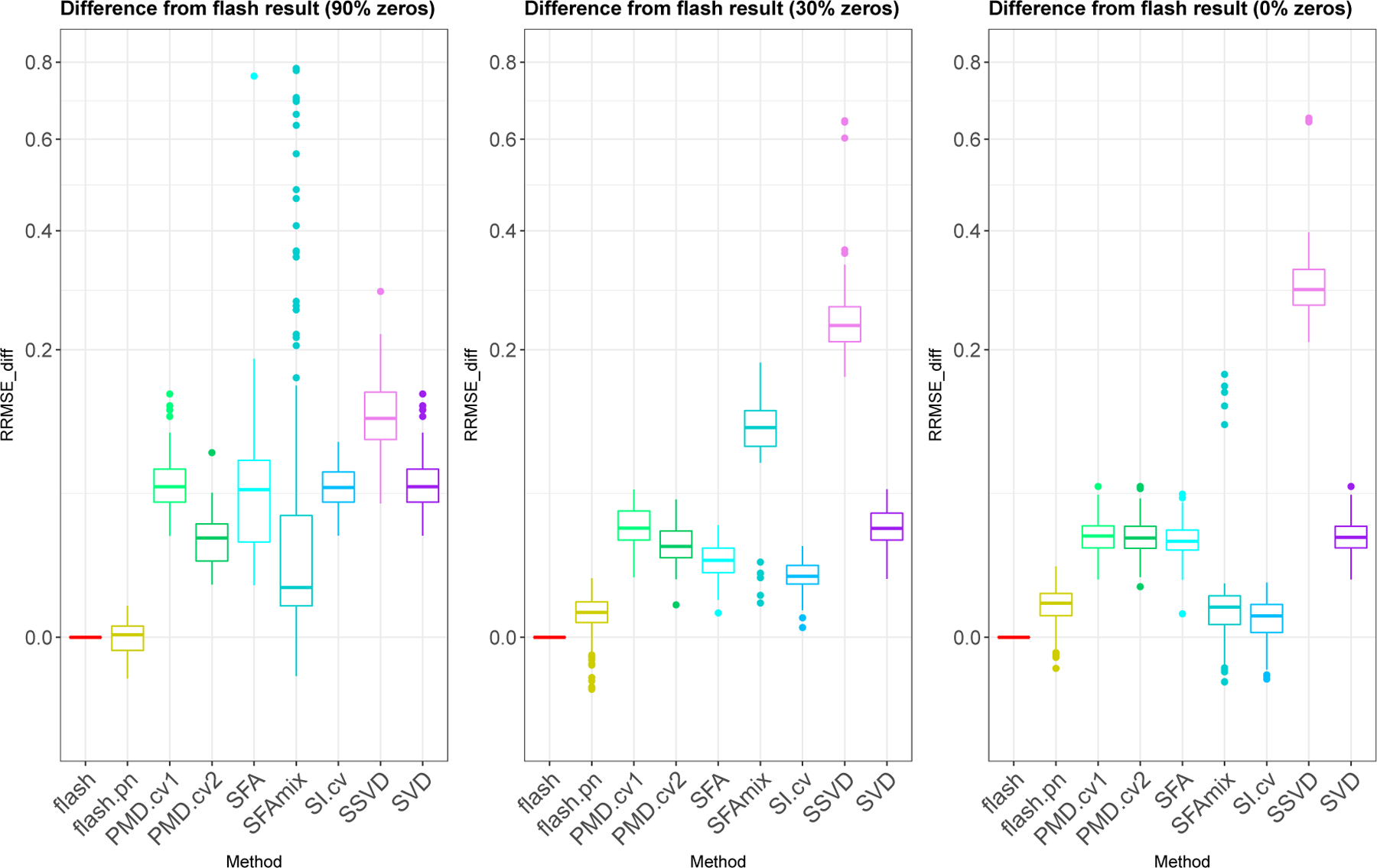
Boxplots comparing accuracy of *flash* with several other methods in a simple rank-1 simulation. This simulation involves a single dense factor, and a loading that varies from strong sparsity (90% zeros, left) to no sparsity (right). Accuracy is measured by difference in each methods RRMSE from the *flash* RRMSE, with smaller values indicating highest accuracy. The y axis is plotted on a nonlinear (square-root) scale to avoid the plots being dominated by poorer-performing methods.

Despite the simplicity of this simulation, the methods vary greatly in performance (Figure 1). Both versions of flash consistently outperform all the other methods across all scenarios (although softImpute performs similarly in the non-sparse case). The next best performances come from softImpute (SI.cv), PMD.cv2 and SFA, whose relative performances depend on the scenario. All three consistently improve on, or do no worse than, SVD. PMD.cv1 performs similarly to SVD. The SFAmix method performs very variably, sometimes providing very poor estimates, possibly due to poor convergence of the MCMC algorithm (it is the only method here that uses MCMC). The SSVD method consistently performs worse than simple SVD, possibly because it is more adapted to both factors and loadings being sparse (and possibly because, following Yang et al. 2014, we did not use CV to tune its parameters). Inspection of individual results suggests that the poor performance of both SFAmix and SSVD is often due to over-shrinking of non-zero loadings to zero.

#### 5.1.2. A Sparse Bi-cluster Example (Rank 3)

An important feature of our EBMF methods is that they estimate separate distributions $g_{l},g_{f}$ for each factor and each loading, allowing them to adapt to any combination of sparsity in the factors and loadings. This flexibility is not easy to achieve in other ways. For example, methods that use CV are generally limited to one or two tuning parameters because of the computational difficulties of searching over a larger space.

**Figure 2:**
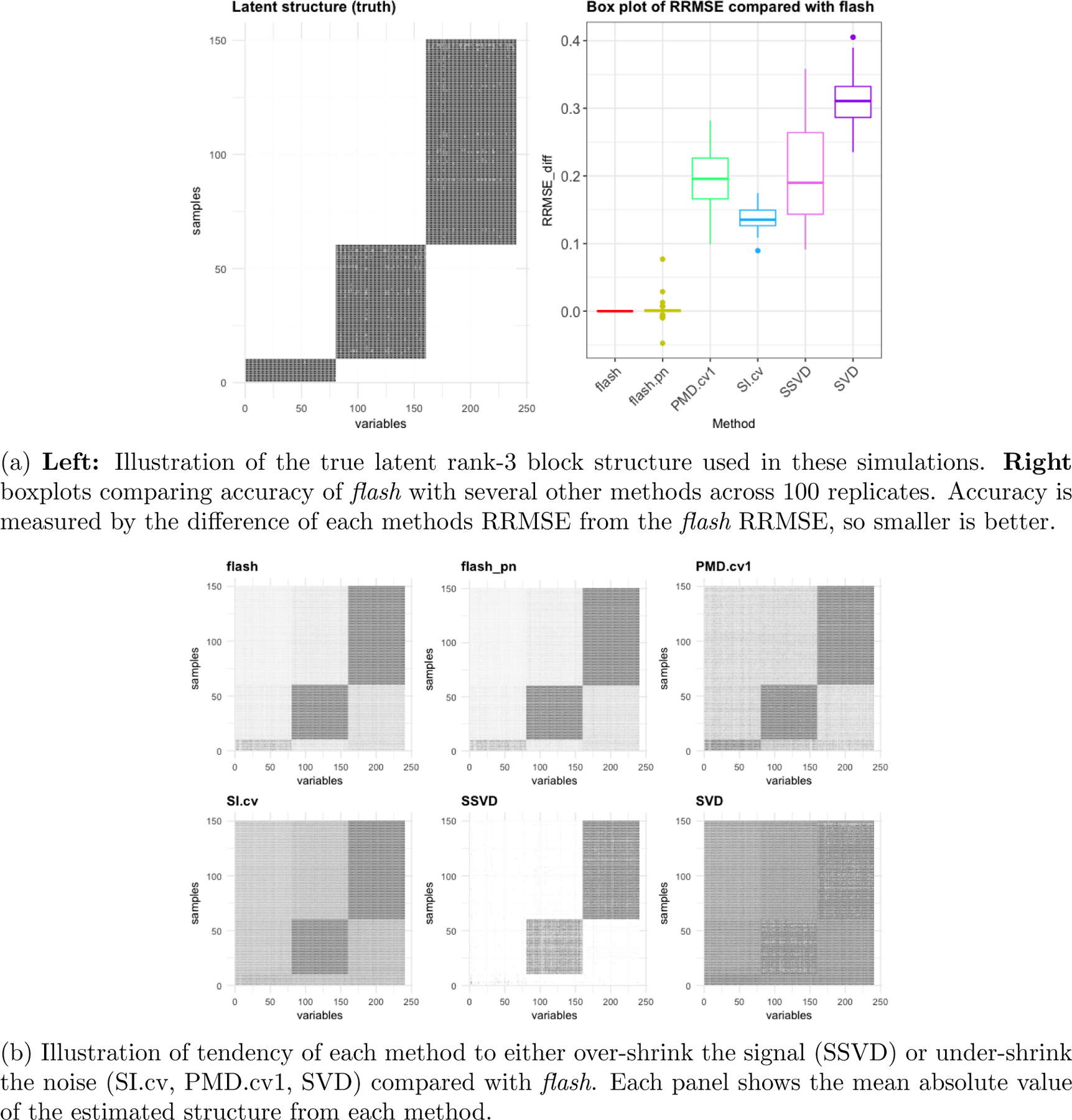
Results from simulations with sparse bi-cluster structure (K=3).

To illustrate this flexibility we simulated data under the factor model (2.1) with = 150,p=240,K=3,τ=1/4, and:

(5.4) $$l_{1,i}\sim N\left(0,2^{2}\right)\;i=1,\ldots ,10$$

(5.5) $$l_{2,i}\sim N(0,1)\;i=11,\ldots ,60$$

(5.6) $$l_{3,i}\sim N\left(0,1/2^{2}\right)\;i=61,\ldots ,150$$

(5.7) $$f_{1,j}\sim N\left(0,1/2^{2}\right)\;j=1,\ldots ,80$$

(5.8) $$f_{2,j}\sim N(0,1)\;j=81,\ldots ,160$$

(5.9) $$f_{3,j}\sim N\left(0,2^{2}\right)\;j=161,\ldots ,240,$$

with all other elements of $l_{k}$ and $f_{k}$ set to zero for k=1,2,3. This example has a sparse bi-cluster structure where distinct groups of samples are each loaded on only one factor (Figure 2a), and both the size of the groups and number of variables in each factor vary.

We applied *flash*, softImpute, SSVD and PMD to this example. (We excluded SFA and SFAmix since these methods do not model sparsity in both factors and loadings.) The results (Figure 2) show that again flash consistently outperforms the other methods, and again the next best is softImpute. On this example both SSVD and PMD outperform SVD. Although SSVD and PMD perform similarly on average, their qualitative behavior is different: PMD insufficiently shrink the 0 values, whereas SSVD shrinks the 0 values well but overshrinks some of the signal, essentially removing the smallest of the three loading/factor combinations (Figure 2 b).

### 5.2. Missing Data Imputation for Real Data Sets

Here we compare methods in their ability to impute missing data using five real data sets. In each case we “hold out” (mask) some of the data points, and then apply the methods to obtain estimates of the missing values. The data sets are as follows:

*MovieLens 100K data*, an (incomplete) 943×1682 matrix of user-movie ratings (integers from 1 to 5) (Harper and Konstan, 2016). Most users do not rate most movies, so the matrix is sparsely observed (94% missing), and contains about 100K observed ratings. We hold out a fraction of the observed entries and assess accuracy of methods in estimating these. We centered and scaled the ratings for each user before analysis.

GTEx eQTL *summary data*, a 16069×44 matrix of Z scores computed testing association of genetic variants (rows) with gene expression in different human tissues (columns). These data come from the Genotype Tissue Expression (GTEx) project (Consortium et al., 2015), which assessed the effects of thousands of “eQTLs” across 44 human tissues. (An eQTL is a genetic variant that is associated with expression of a gene.) To identify eQTLs, the project tested for association between expression and every near-by genetic variant, each test yielding a Z score. The data used here are the Z scores for the most significant genetic variant for each gene (the “top” eQTL). See Section 5.3 for more detailed analyses of these data.

*Brain Tumor data*, a 43×356 matrix of gene expression measurements on 4 different types of brain tumor (included in the denoiseR package, Josse et al., 2018). We centered each column before analysis.

*Presidential address data*, a 13×836 matrix of word counts from the inaugural addresses of 13 US presidents (1940–2009) (also included in the denoiseR package, Josse et al., 2018). Since both row and column means vary greatly we centered and scaled both rows and columns before analysis, using the biScale function from softImpute.

*Breast cancer data*, a 251×226 matrix of gene expression measurements from Carvalho et al. (2008), which were used as an example in the paper introducing NBSFA (Knowles and Ghahramani, 2011). Following Knowles and Ghahramani (2011) we centered each column (gene) before analysis.

Among the methods considered above, only *flash*, PMD and softImpute can handle missing data. We add NBSFA (Knowles and Ghahramani, 2011) to these comparisons. To emphasize the importance of parameter tuning we include results for PMD and softImpute with default settings (denoted PMD, SI) as well as using cross-validation (PMD.cv1, SI.cv).

For these real data the appropriate value of K is, of course, unknown. Both *flash* and NBSFA automatically estimate K. For PMD and softImpute we specified K based on the values inferred by *flash* and NBSFA. (Specifically, we used K=10,30,20,10,40 respectively for the five data sets.)

We applied each method to all 5 data sets, using 10-fold OCV (Appendix B) to mask data points for imputation, repeated 20 times (with different random number seeds) for each data set. We measure imputation accuracy using root mean squared error (RMSE):

(5.10) $$\text{RMSE}(\overset{ˆ}{Y},Y;\Omega )=\sqrt{\frac{1}{\left|\Omega \right|}\sum_{ij\in \Omega }\;\;{\left(Y_{ij}-{\overset{ˆ}{Y}}_{ij}\right)}^{2}}.$$

where Ω is the set of indices of the held-out data points.

**Figure 3:**
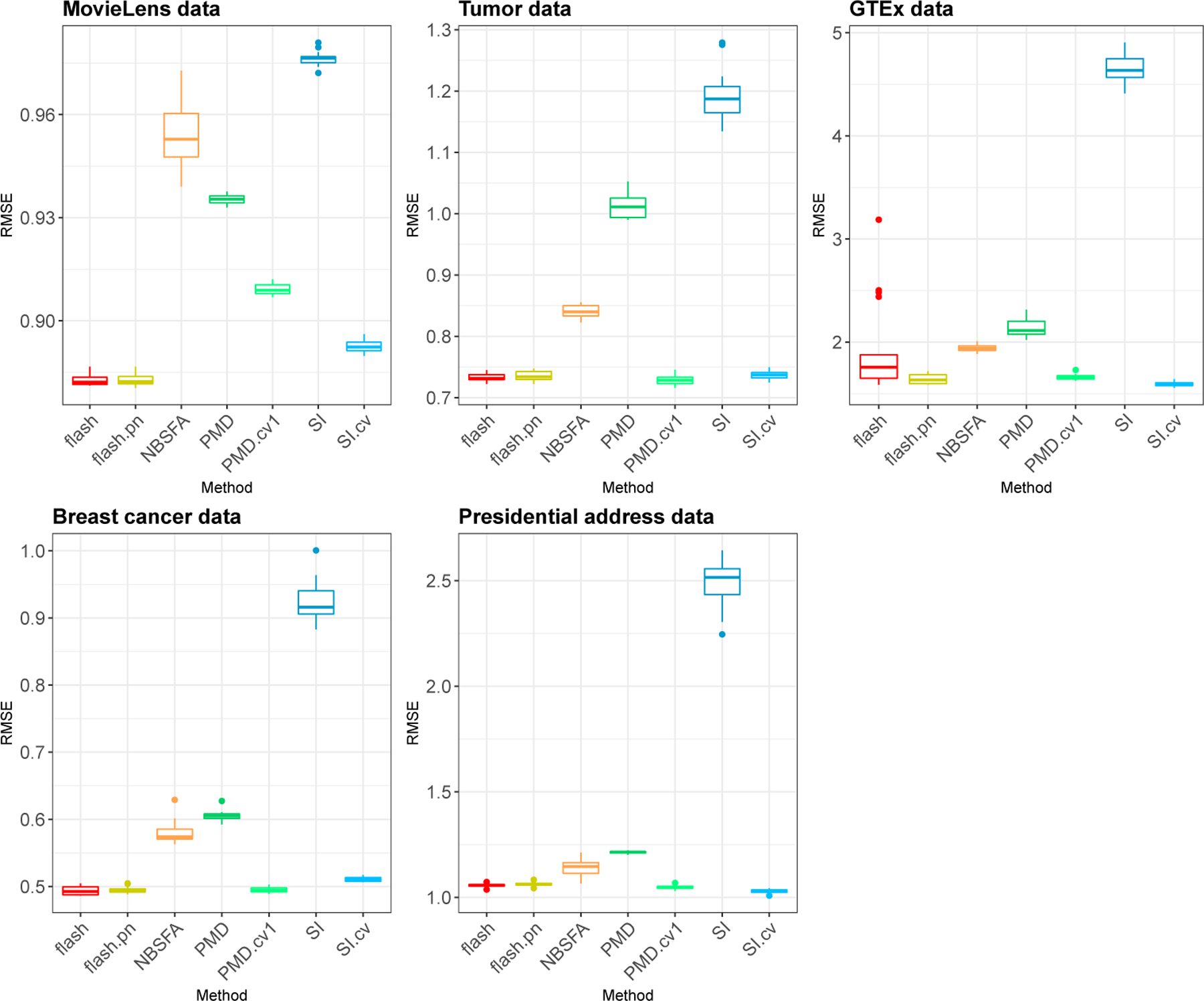
Comparison of the accuracy of different methods in imputing missing data. Each panel shows a boxplot of error rates (RMSE) for 20 simulations based on masking observed entries in a real data set.

The results are shown in Figure 3. Although the ranking of methods varies among data sets, *flash*, PMD.cv1 and SI.cv perform similarly on average, and consistently outperform NBSFA, which in turn typically outperforms (untuned) PMD and unpenalized softImpute. These results highlight the importance of appropriate tuning for the penalized methods, and also the effectiveness of the EB method in *flash* to provide automatic tuning.

In these comparisons, as in the simulations, the two *flash* methods typically performed similarly. The exception is the GTEx data, where the scale mixture of normals (𝒢=𝒮N) performed worse. Detailed investigation revealed this to be due to a very small number of very large “outlier” imputed values, well outside the range of the observed data, which grossly inflated RMSE. These outliers were so extreme that it should be possible to implement a filter to avoid them. However, we did not do this here as it seems useful to highlight this unexpected behavior. (Note that this occurs only when data are missing, and even then only in one of the five data sets considered here.)

### 5.3. Sharing of Genetic Effects on Gene Expression Among Tissues

To illustrate *flash* in a scientific application, we applied it to the GTEx data described above, a 16,069×44 matrix of Z scores, with $Z_{ij}$ reflecting the strength (and direction) of effect of eQTL i in tissue j. We applied flash with 𝒢=𝒮N using the greedy+backfitting algorithm (i.e. the backfitting algorithm, initialized using the greedy algorithm).

**Figure 4:**
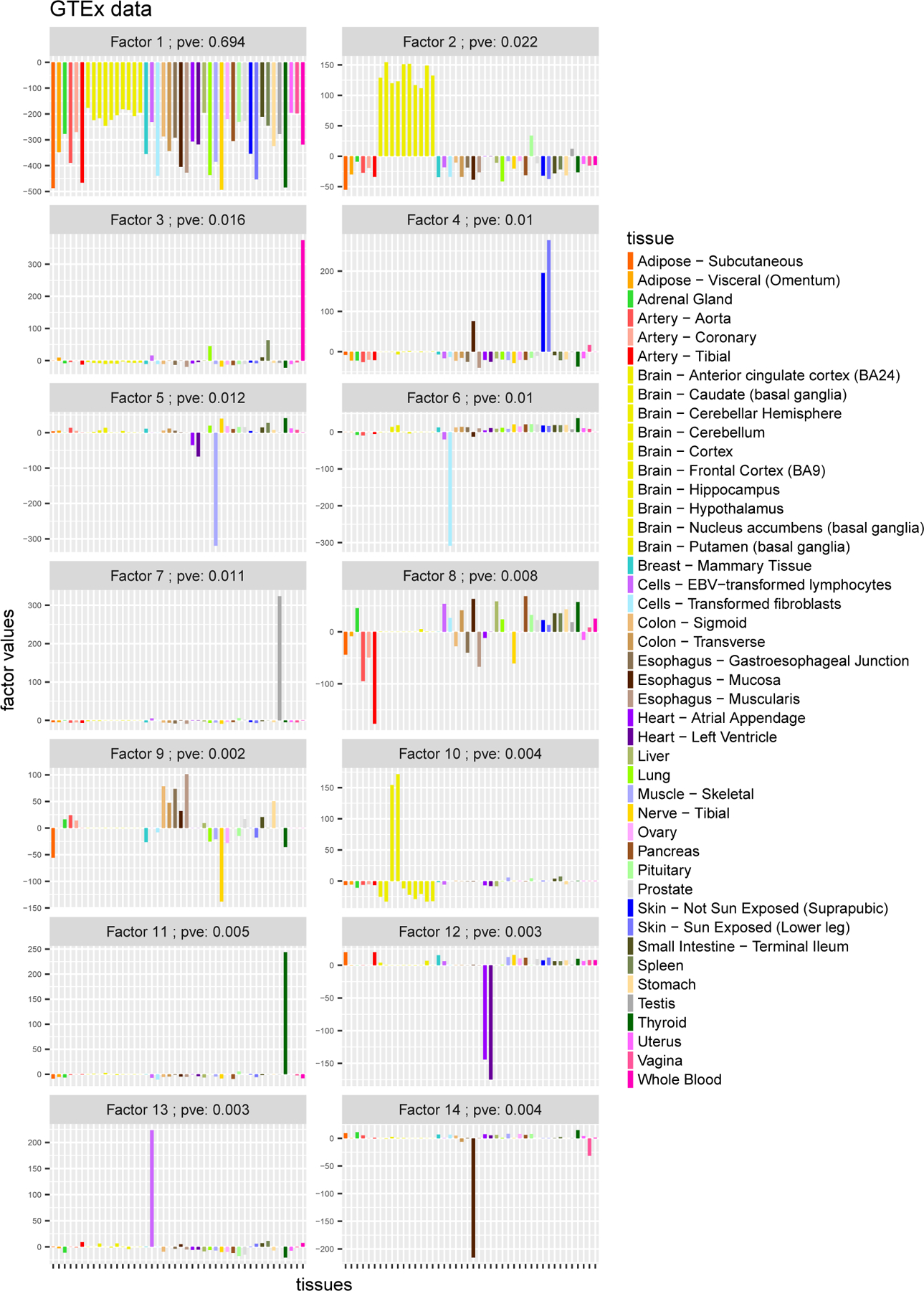
Results from running flash on GTEx data (factors 1 – 8). The pve (“Percentage Variance Explained”) for loading/factor k is defined as ${\text{pve}}_{k}≔s_{k}/\left(\sum_{k}\;s_{k}+\right.\left.\sum_{ij}\;1/\tau_{ij}\right)$ where $s_{k}≔\sum_{ij}\;{\left({\overset{-}{l}}_{ki}{\overset{-}{f}}_{kj}\right)}^{2}$. It is a measure of the amount of signal in the data captured by loading/factor k (but its naming as “percentage variance explained” should be considered loose since the factors are not orthogonal).

**Figure 5:**
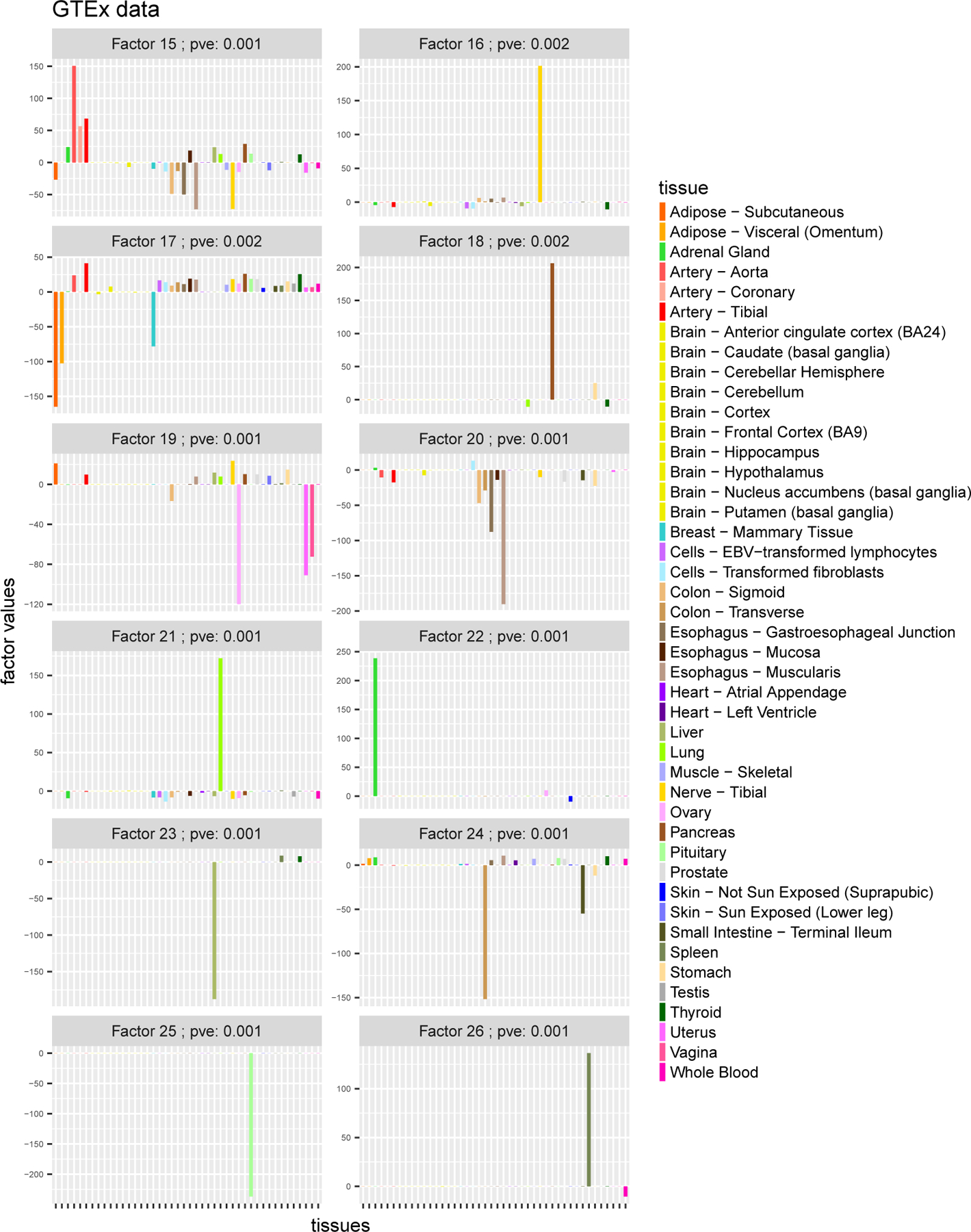
Results from running *flash* on GTEx data (factors 15 – 26)

The *flash* results yielded 26 factors (Figure 4–5) which summarize the main patterns of eQTL sharing among tissues (and, conversely, the main patterns of tissue-specificity). For example, the first factor has approximately equal weight for every tissue, and reflects the fact that many eQTLs show similar effects across all 44 tissues. The second factor has strong effects only in the 10 brain tissues, from which we infer that some eQTLs show much stronger effects in brain tissues than other tissues.

Subsequent factors tend to be sparser, and many have a strong effect in only one tissue, capturing “tissue-specific” effects. For example, the 3rd factor shows a strong effect only in whole blood, and captures eQTLs that have much stronger effects in whole blood than other tissues. (Two tissues, “Lung” and “Spleen”, show very small effects in this factor but with the same sign as blood. This is intriguing since the lung has recently been found to make blood cells—see Lefrançais et al. 2017—and a key role of the spleen is storing of blood cells.) Similarly Factors 7, 11 and 14 capture effects specific to “Testis”, “Thyroid” and “Esophagus Mucosa” respectively.

A few other factors show strong effects in a small number of tissues that are known to be biologically related, providing support that the factors identified are scientifically meaningful. For example, factor 10 captures the two tissues related to the cerebellum, “Brain Cerebellar Hemisphere” and “Brain Cerebellum”. Factor 19 captures tissues related to female reproduction, “Ovary”, “Uterus” and “Vagina”. Factor 5 captures “Muscle Skeletal”, with small but concordant effects in the heart tissues (“Heart Atrial Appendage” and “Heart Left Ventricle”). Factor 4, captures the two skin tissues (“Skin Not Sun Exposed Suprapubic”, “Skin Sun Exposed Lower leg”) and also “Esophagus Mucosa”, possibly reflecting the sharing of squamous cells that are found in both the surface of the skin, and the lining of the digestive tract. In factor 24, “Colon Transverse” and “Small Intestine Terminal Ileum” show the strongest effects (and with same sign), reflecting some sharing of effects in these intestinal tissues. Among the 26 factors, only a few are difficult to interpret biologically (e.g. factor 8).

**Figure 6:**
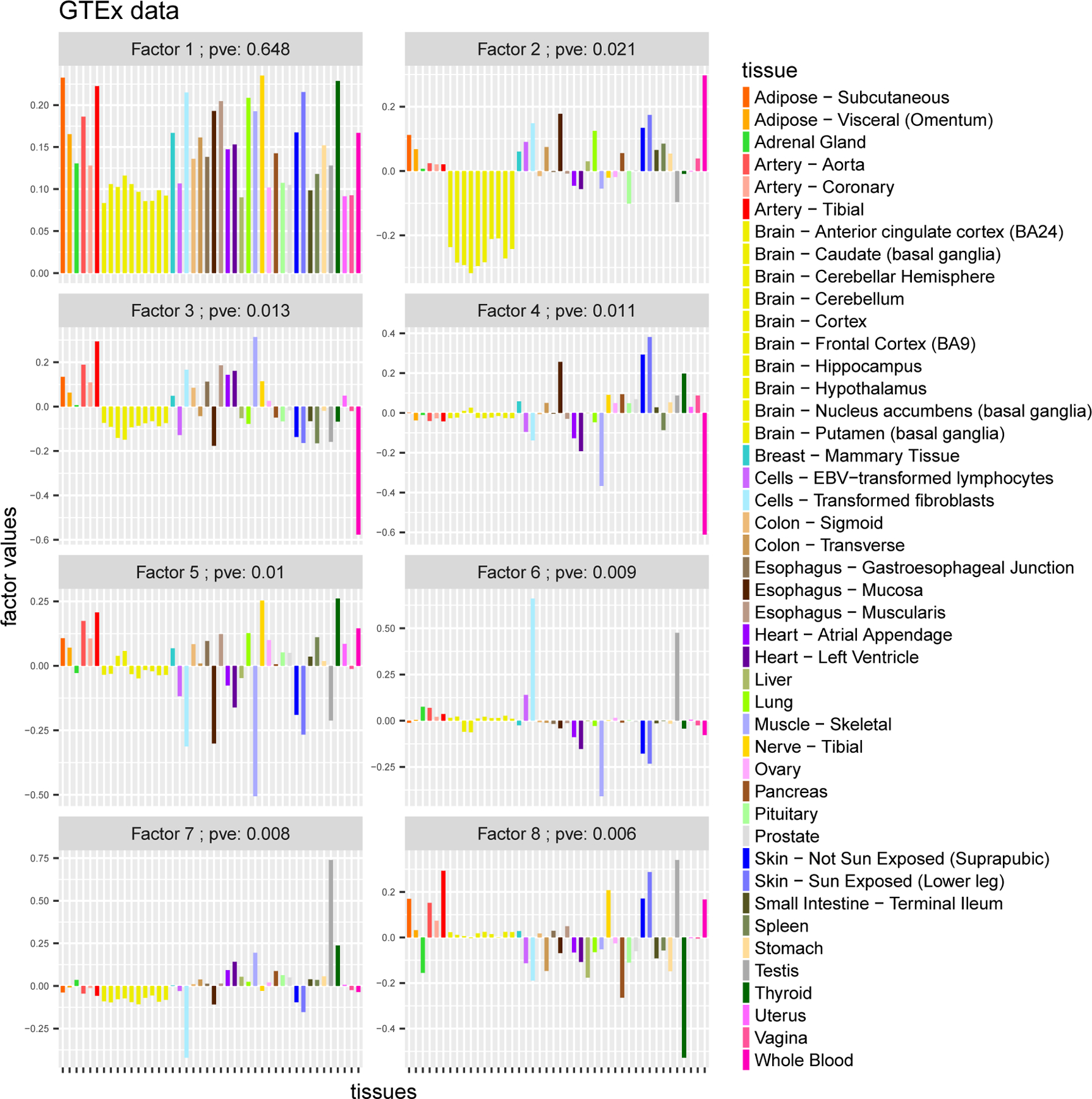
Results from running softImpute on GTEx data (factors 1–8). The factors are both less sparse and less interpretable than the *flash* results.

To highlight the benefits of sparsity, we contrast the *flash* results with those for softImpute, which was the best-performing method in the missing data assessments on these data, but which uses a nuclear norm penalty that does not explicitly reward sparse factors or loadings. The first eight softImpute factors are shown in Figure 6. The softImpute results—except for the first two factors—show little resemblance to the flash results, and in our view are harder to interpret.

### 5.4. Computational Demands

It is difficult to make general statements about computational demands of our methods, because both the number of factors and number of iterations per factor can vary considerably depending on the data. However, to give a specific example, running our current implementation of the greedy algorithm on the GTEx data (a 16,000 by 44 matrix) takes about 140s (wall time) for 𝒢=𝒫N and 650s for 𝒢=𝒮N (on a 2015 MacBook Air with a 2.2 GHz Intel Core i7 processor and 8Gb RAM). By comparison, a single run of softImpute without CV takes 2–3s, so a naive implementation of 5-fold CV with 10 different tuning parameters and 10 different values of K would take over 1000s (although one could improve on this by use of warm starts for example).

## 6. Discussion

Here we discuss some potential extensions or modifications of our work.

### 6.1. Orthogonality Constraint

Our formulation here does not require the factors or loadings to be orthogonal. In scientific applications we do not see any particular reason to expect underlying factors to be orthogonal. However, imposing such a constraint could have computational or mathematical advantages. Formally adding such a constraint to our objective function seems tricky, but it would be straightforward to modify our algorithms to include an orthogonalization step each update. This would effectively result in an EB version of the SSVD algorithms in Yang et al. (2014), and it seems likely to be computationally faster than our current approach. One disadvantage of this approach is that it is unclear what optimization problem such an algorithm would solve (but the same is true of SSVD, and our algorithms have the advantage that they deal with missing data.)

### 6.2. Non-negative Matrix Factorization

We focused here on the potential for EBMF to induce sparsity on loadings and factors. However, EBMF can also encode other assumptions. For example, to assume the loadings and factors are non-negative, simply restrict 𝒢 to be a family of non-negative-valued distributions, yielding “Empirical Bayes non-negative Matrix Factorization” (EBNMF). Indeed, the ashr software can already solve the EBNM problem for some such families 𝒢, and so *flash* already implements EBNMF. In preliminary assessments we found that the greedy approach is problematic here: the non-negative constraint makes it harder for later factors to compensate for errors in earlier factors. However, it is straightforward to apply the backfitting algorithm to fit EBNMF, with initialization by any existing NMF method. The performance of this approach is an area for future investigation.

### 6.3. Tensor Factorization

It is also straightforward to extend EBMF to tensor factorization, specifically a CANDE-COMP/PARAFAC decomposition (Kolda and Bader, 2009):

(6.1) $$Y_{ijm}=\sum_{k=1}^{K}\;\;l_{ki}f_{kj}h_{km}+E_{ijm}$$

(6.2) $$l_{k1},\ldots ,l_{kn}\sim^{iid}g_{l_{k}},\;g_{l_{k}}\in 𝒢$$

(6.3) $$f_{k1},\ldots ,f_{kp}\sim^{iid}g_{f_{k}},\;g_{f_{k}}\in 𝒢$$

(6.4) $$h_{k1},\ldots ,h_{kr}\sim {\;}^{iid}g_{h_{k}},\;g_{h_{k}}\in 𝒢$$

(6.5) $$E_{ijm}\sim^{iid}N\left(0,1/\tau_{ijm}\right).$$

The variational approach is easily extended to this case (a generalization of methods in Hore et al., 2016), and updates that increase the objective function can be constructed by solving an EBNM problem, similar to EBMF. It seems likely that issues of convergence to local optima, and the need for good initializations, will need some attention to obtain good practical performance. However, results in Hore et al. (2016) are promising, and the automatic-tuning feature of EB methods seems particularly attractive here. For example, extending PMD to this case—allowing for different sparsity levels in l,f and h—would require 3 penalty parameters even in the rank 1 case, making it difficult to tune by CV.

### 6.4. Non-Gaussian Errors

It is also possible to extend the variational approximations used here to fit non-Gaussian models, such as binomial data; see for example Jaakkola and Jordan (2000); Seeger and Bouchard (2012); Klami (2015). The extension of our EB methods using these ideas is detailed in Wang (2017).

## Appendix A. Variational EBMF with K Factors

Here we describe in detail the variational approach to the K factor model, including deriving updates that we use to optimize the variational objective. (These derivations naturally include the K=1 model as a special case, and our proof of Proposition 5 below includes Proposition 2 as a special case.)

Let $q_{l},q_{f}$ denote the variational distributions on the K loadings/factors:

(A.1)
$$q_{l}\left(l_{1},\cdots ,l_{K}\right)\;=\prod_{k}\;\;q_{l_{k}}\left(l_{k}\right)$$

(A.2)
$$\begin{array}{rr} q_{f}\left(f_{1},\ldots ,f_{K}\right) & \;=\prod_{k}\;\;q_{f_{k}}\left(f_{k}\right) \end{array}.$$

The objective function F(3.4) is thus a function of $q_{l}=\left(q_{l_{1}},\ldots ,q_{l_{K}}\right),q_{f}=\left(q_{f_{1}},\ldots ,q_{f_{K}}\right)$, $g_{l}=\left(g_{l_{1}},\ldots ,g_{l_{K}}\right)$ and $g_{f}=\left(g_{f_{1}},\ldots ,g_{f_{K}}\right)$, as well as the precision τ:

(A.3)
$$F\left(q_{l},q_{f},g_{l},g_{f},\tau \right)=\int \;\underset{k}{\prod }q_{l_{k}}\left(l_{k}\right)q_{f_{k}}\left(f_{k}\right)\text{log}\frac{p\left(Y,l,f;g_{l_{1}},g_{f_{1}},\cdots ,g_{l_{K}},g_{f_{K}},\tau \right)}{\prod_{k}q_{l_{k}}(l_{k})q_{f_{k}}\left(f_{k}\right)}dl_{k}df_{k},$$

(A.4)
$$=E_{q_{l},q_{f}}\text{log}p(Y|l,f;\tau )+\sum_{k}\;\;E_{q_{l_{k}}}\text{log}\frac{g_{l_{k}}\left(l_{k}\right)}{q_{l_{k}}\left(l_{k}\right)}+\sum_{k}\;\;E_{q_{f_{k}}}\text{log}\frac{g_{f_{k}}\left(f_{k}\right)}{q_{f_{k}}\left(f_{k}\right)}.$$

We optimize F by iteratively updating parameters relating to τ, a single loading k $\left(q_{l_{k}},g_{l_{k}}\right)$ or factor $k\left(q_{f_{k}},g_{f_{k}}\right)$, keeping other parameters fixed. We simplify implementation by keeping track of only the first and second moments of the distributions $q_{l_{k}}$ and $q_{f_{k}}$, which we denote ${\overset{-}{l}}_{k},{\bar{l^{2}}}_{k},{\overset{-}{f}}_{k},{\overset{-}{f^{2}}}_{k}$. We now describe each kind of update in turn.

### A.1 Updates for Precision Parameters

Here we derive updates to optimize F over the precision parameters τ. Focusing on the parts of F that depend on τ gives:

(A.5)
$$F\left(\tau \right)=E_{q_{l}}E_{q_{f}}\underset{ij}{\sum }0.5\text{log}\left(\tau_{ij}\right)-0.5\tau_{ij}{\left(Y_{ij}-\underset{k}{\sum }l_{ki}f_{kj}\right)}^{2}+\text{const}$$

(A.6)
$$=0.5\sum_{ij}\;\;\left[\text{log}\left(\tau_{ij}\right)+\tau_{ij}{\bar{R^{2}}}_{ij}\right]+\text{const}$$

where $\overset{-}{R^{2}}$ is defined by:

(A.7)
$${\bar{R^{2}}}_{ij}≔E_{q_{l},q_{f}}\left[{\left(Y_{ij}-\overset{K}{\underset{k=1}{\sum }}l_{ki}f_{kj}\right)}^{2}\right]$$

(A.8)
$$={\left(Y_{ij}-\underset{k}{\sum }{\bar{l}}_{ki}{\bar{f}}_{kj}\right)}^{2}-\underset{k}{\sum }{\left({\bar{l}}_{ki}\right)}^{2}{\left({\bar{f}}_{kj}\right)}^{2}+\underset{k}{\sum }{\bar{l^{2}}}_{ki}{\bar{f^{2}}}_{kj}.$$

If we constrain τ∈𝒯 then we have

(A.9)
$$\hat{\tau }=\text{arg}\underset{\tau \in 𝒯}{\text{max}}\underset{ij}{\sum }\left[\text{log}\left(\tau_{ij}\right)-\tau_{ij}{\bar{R^{2}}}_{ij}\right].$$

For example, assuming constant precision $\tau_{ij}=\tau$ yields:

(A.10)
$$\hat{\tau }=\frac{NP}{\sum_{ij}{\bar{R^{2}}}_{ij}}.$$

Assuming column-specific precisions $\left(\tau_{ij}=\tau_{j}\right)$, which is the default in our software, yields:

(A.11)
$${\hat{\tau }}_{j}=\frac{N}{\sum_{i}{\bar{R^{2}}}_{ij}}.$$

Other variance structures are considered in Appendix A.5 of Wang (2017).

### A.2 Updating Loadings and Factors

The following Proposition, which generalizes Proposition 2 in the main text, shows how updates for loadings (and factors) for the K-factor EBMF model can be achieve by solving an EBNM problem.

**Proposition 5** *For the* K-*factor model*, arg ${\text{max}}_{q_{l_{k}},g_{l_{k}}}\;F\left(q_{l},q_{f},g_{l},g_{f},\tau \right)$ *is solved by solving an EBNM problem. Specifically*

(A.12)
$$\text{arg}\underset{q_{l_{k}},g_{l_{k}}}{\text{max}}F\left(q_{l},q_{f},g_{l},g_{f},\tau \right)=\text{EBNM}\left(\hat{l}\left(R^{k},{\bar{f}}_{k},{\bar{f^{2}}}_{k},\tau \right),s_{l}\left({\bar{f^{2}}}_{k},\tau \right)\right)$$

*where the functions* $\overset{ˆ}{l}$ *and* $s_{l}$ *are given by* (3.19) *and* (3.20), ${\overset{-}{f}}_{k},{\overset{-}{f^{2}}}_{k}\in R^{p}$ *denote the vectors whose elements are the first and second moments of* $f_{k}$ *under* $q_{f_{k}}$, *and* $R^{k}$ *denotes the residual matrix* (3.27).

*Similarly*, $\text{arg}{\text{max}}_{q_{f_{k}},g_{f_{k}}}\;F\left(q_{l},q_{f},g_{l},g_{f},\tau \right)$ *is solved by solving an EBNM problem. Specifically*,

(A.13)
$$\text{arg}\underset{q_{f_{k}},g_{f_{k}}}{\text{max}}F\left(q_{l},q_{f},g_{l},g_{f},\tau \right)=\text{EBNM}\left(\hat{f}\left(R^{k},{\bar{l}}_{k},{\bar{l^{2}}}_{k},\tau \right),s_{f}\left({\bar{l^{2}}}_{k},\tau \right)\right)$$

*where the functions* $\overset{ˆ}{f}:R^{n\times p}\times R^{n}\times R^{n}\times R^{n\times p}\to R^{p}$ *and* $s_{f}:R^{n}\times R^{n\times p}\to R^{p}$ *are given by*

$$\begin{array}{rr} \overset{ˆ}{f}(Y,v,w,\tau {)}_{j} & \;≔\frac{\sum_{i}\;\;\tau_{ij}Y_{ij}v_{i}}{\sum_{i}\;\;\tau_{ij}w_{i}}, \end{array}$$

(A.15)
$$s_{f}(w,\tau {)}_{j}\;≔{\left(\sum_{i}\;\;\tau_{ij}w_{i}\right)}^{-0.5}.$$

#### A.2.1 A Lemma on the Normal Means Problem

To prove Proposition 5 we introduce a lemma that characterizes the solution of the normal means problem in terms of an objective that is closely related to the variational objective.

Recall that the EBNM model is:

(A.16)
x=θ+e

(A.17)
$$\begin{array}{c} \theta_{1},\ldots ,\theta_{n}\sim^{iid}g,\;g\in 𝒢 \end{array}.$$

where $e_{i}\sim N\left(0,s_{i}^{2}\right)$.

Solving the EBNM problem involves estimating g by maximum likelihood:

(A.18)
$$\overset{ˆ}{g}=\text{arg}\underset{g\in 𝒢}{\text{max}}\;l(g),$$

where

(A.19)
l(g)=log⁡p(x∣g).

It also involves finding the posterior distributions:

(A.20)
$$p(\theta |x,\overset{ˆ}{g})=\prod_{j}\;p\left(\theta_{j}|x,\overset{ˆ}{g}\right)\propto \prod_{j}\;\overset{ˆ}{g}\left(\theta_{j}\right)p\left(x_{j}|\theta_{j},s_{j}\right).$$

**Lemma 6** *Solving the EBNM problem also solves*:

(A.21)
$$\underset{q_{\theta },g\in 𝒢}{\text{max}}\;F^{NM}\left(q_{\theta },g\right)$$

*where*

(A.22)
$$F^{NM}\left(q_{\theta },g\right)=E_{q_{\theta }}\left[-\frac{1}{2}\sum_{j}\;\;\left(A_{j}\theta_{j}^{2}-2B_{j}\theta_{j}\right)\right]+E_{q_{\theta }}\text{log}\frac{g(\theta )}{q_{\theta }(\theta )}+\text{const}$$

*with* $A_{j}=1/s_{j}^{2}$ *and* $B_{j}=x_{j}/s_{j}^{2}$, *and* $g(\theta )≔\prod_{j}\;g\left(\theta_{j}\right)$.

*Equivalently*, (A.21)-(A.22) *is solved by* $g=\overset{ˆ}{g}$ in (A.18) *and* $q_{\theta }=p(\theta |x,\overset{ˆ}{g})$ *in* (A.20), *with* $x_{j}=B_{j}/A_{j}$ *and* $s_{j}^{2}=1/A_{j}$.

**Proof** The log likelihood can be written as

(A.23)
$$l\left(g\right)≔\text{log}[p(x|g)]$$

(A.24)
=log⁡[p(x,θ∣g)/p(θ∣x,g)]

(A.25)
$$=\int \;q_{\theta }(\theta )\text{log}\frac{p(x,\theta |g)}{p(\theta |x,g)}d\theta$$

(A.26)
$$=\int \;q_{\theta }(\theta )\text{log}\frac{p(x,\theta |g)}{q_{\theta }(\theta )}d\theta +\int \;q_{\theta }(\theta )\text{log}\frac{q_{\theta }(\theta )}{p(\theta |x,g)}d\theta$$

(A.27)
$$=F^{\text{NM}}\left(q_{\theta },g\right)+D_{KL}\left(q_{\theta }∥p_{\theta |x,g}\right)$$

where

(A.28)
$$F^{\text{NM}}\left(q_{\theta },g\right)=\int \;q_{\theta }(\theta )\text{log}\frac{p(x,\theta |g)}{q_{\theta }(\theta )}d\theta$$

and

(A.29)
$$D_{KL}\left(q_{\theta }||p_{\theta |x,g}\right)=-\int \;q_{\theta }(\theta )\text{log}\frac{p(\theta |x,g)}{q_{\theta }(\theta )}d\theta$$

Here $p_{\theta |x,g}$ denotes the posterior distribution p(θ∣x,g). This identity holds for any distribution $q_{\theta }(\theta )$.

Rearranging (A.27) gives:

(A.30)
$$F^{\text{NM}}\left(q_{\theta },g\right)=l(g)-D_{KL}\left(q_{\theta }∥p_{\theta |x,g}\right).$$

Since $D_{KL}\left(q_{\theta }||p_{\theta |x,g}\right)\geq 0$, with equality when $q_{\theta }=p_{\theta |x,g},F^{\text{NM}}\left(q_{\theta },g\right)$ is maximized over $q_{\theta }$ by setting $q_{\theta }=p_{\theta |x,g}$. Further

(A.31)
$$\underset{q_{\theta }}{\text{max}}\;F^{\text{NM}}\left(q_{\theta },g\right)=l(g),$$

so

(A.32)
$$\text{arg}\underset{g\in 𝒢}{\text{max}}\;\underset{q_{\theta }}{\text{max}}\;F^{\text{NM}}\left(q_{\theta },g\right)=\text{arg}\underset{g\in 𝒢}{\text{max}}\;l(g)=\overset{ˆ}{g}.$$

It remains only to show that $F^{\text{NM}}$ has the form (A.22).

By (A.16) and (A.17), we have

(A.33)
$$\text{log}p(x,\theta |g)=-\frac{1}{2}\sum_{j}\;s_{j}^{-2}{\left(x_{j}-\theta_{j}\right)}^{2}+\text{log}g(\theta )+\text{const}.$$

Thus

(A.34)
$$F^{\text{NM}}\left(q_{\theta },g\right)=E_{q_{\theta }}\left[-\frac{1}{2}\sum_{j}\;\;\left(A_{j}\theta_{j}^{2}-2B_{j}\theta_{j}\right)\right]+{\text{E}}_{q_{\theta }}\text{log}\frac{g(\theta )}{q_{\theta }(\theta )}+\text{const}.$$

■

#### A.2.2 Proof of Proposition 5

We are now ready to prove Proposition 5.

**Proof** We prove the first part of the proposition since the proof for the second part is essentially the same.

The objective function (A.3) is:

(A.35)
$$F\left(q_{l},q_{f},g_{l},g_{f},\tau \right)=E_{q_{l},q_{f}}\text{log}p\left(Y|l,f;\tau \right)+\underset{k}{\sum }E_{q_{l_{k}}}\text{log}\frac{g_{l_{k}}\left(l_{k}\right)}{q_{l_{k}}\left(l_{k}\right)}+\underset{k}{\sum }E_{q_{f_{k}}}\text{log}\frac{g_{f_{k}}\left(f_{k}\right)}{q_{f_{k}}\left(f_{k}\right)}$$

(A.36)
$$=E_{q_{l_{k}}}\left[-\frac{1}{2}\sum_{i}\;\;\left(A_{ik}l_{ki}^{2}-2B_{ik}l_{ki}\right)\right]+E_{q_{l_{k}}}\text{log}\frac{g_{l_{k}}\left(l_{k}\right)}{q_{l_{k}}\left(l_{k}\right)}+C_{1}$$

where $C_{1}$ is a constant with respect to $q_{l_{k}},g_{l_{k}}$ and

(A.37)
$$A_{ik}=\sum_{j}\;\;\tau_{ij}{\text{E}}_{q_{f}}\left(f_{kj}^{2}\right)$$

(A.38)
$$B_{ik}\;=\sum_{j}\;\;\tau_{ij}\left(R_{ij}^{k}{\text{E}}_{q_{f}}f_{kj}\right).$$

Based on Lemma 6, we can solve this optimization problem (A.36) by solving the EBNM problem with:

(A.39)
$$x_{i}=\frac{\sum_{j}\;\;\tau_{ij}\left(R_{ij}^{k}{\text{E}}_{q_{f}}f_{kj}\right)}{\sum_{j}\;\;\tau_{ij}{\text{E}}_{q_{f}}\left(f_{kj}^{2}\right)}$$

(A.40)
$$s_{i}^{2}\;=\frac{1}{\sum_{j}\;\;\tau_{ij}{\text{E}}_{q_{f}}\left(f_{kj}^{2}\right)}.$$

■

### A.3 Algorithms

Just as with the rank 1 EBMF model, the updates for the rank K model require only the first and second moments of the variational distributions q. Thus we implement the updates in algorithms that keep track of the first moments $\left(\overset{-}{l}≔\left({\overset{-}{l}}_{1},\ldots ,{\overset{-}{l}}_{K}\right)\right.$ and $\left.\overset{-}{f}≔\left({\overset{-}{f}}_{1},\ldots ,{\overset{-}{f}}_{K}\right)\right)$ and second moments ($\bar{l^{2}}≔\left({\bar{l^{2}}}_{1},\ldots ,{\bar{l^{2}}}_{K}\right)$ and $\bar{f^{2}}≔\left({\bar{f^{2}}}_{1},\ldots ,{\bar{f^{2}}}_{K}\right)$), and the precision τ.

Algorithm 3 implements a basic update for τ, and for the parameters relating to a single factor $k\left({\bar{l}}_{k},{\bar{l^{2}}}_{K},{\bar{f}}_{k},{\bar{f^{2}}}_{K}\right)$. Note that the latter updates are identical to the updates for fitting the single factor EBMF model, but with $Y_{ij}$ replaced with the residuals obtained by removing the estimated effects of the other k-1 factors.

Based on these basic updates we implemented two algorithms for fitting the K-factor EBMF model: the greedy algorithm, and the backfitting algorithm, as follows.

#### A.3.1 Greedy Algorithm

The greedy algorithm is a forward procedure that, at the kth step, adds new factors and loadings $l_{k},f_{k}$ by optimizing over $q_{l_{k}},q_{f_{k}},g_{l_{k}},g_{f_{k}}$ while keeping the distributions related to previous factors fixed. Essentially this involves fitting the single-factor model to the residuals obtained by removing previous factors. The procedure stops adding factors when the estimated new factors (or loadings) are identically zero. The algorithm as follows:

#### A.3.2 Backfitting Algorithm

The backfitting algorithm iteratively refines a fit of K factors and loadings, by updating them one at a time, at each update keeping the other loadings and factors fixed. The name comes from its connection with the backfitting algorithm in Breiman and Friedman (1985), specifically the fact that it involves iteratively re-fitting to residuals.

### A.4 Objective Function Computation

The algorithms above all involve updates that will increase (or, at least, not decrease) the objective function $F\left(q_{l},q_{f},g_{l},g_{f},\tau \right)$. However, these updates do not require computing the objective function itself. In iterative algorithms it can be helpful to compute the objective function to monitor convergence (and as a check on implementation). In this subsection we describe how this can be done. In essence, this involves extending the solver of the EBNM problem to also return the value of the log-likelihood achieved in that problem (which is usually not difficult).

The objective function of the EBMF model is:

(A.41)
$$F\left(q_{l},q_{f},g_{l},g_{f},\tau \right)=E_{q_{l},q_{f}}\text{log}p(Y|l,f;\tau )+E_{q_{l}}\text{log}\frac{g_{l}(l)}{q_{l}(l)}+E_{q_{f}}\text{log}\frac{g_{f}(f)}{q_{f}(f)}$$

The calculation of $E_{q_{l},q_{f}}\text{log}p(Y|l,f;\tau )$ is straightforward and $E_{q_{l}}\text{log}\frac{g_{l}(l)}{q_{l}(l)}$ and $E_{q_{l}}\text{log}\frac{g_{l}(l)}{q_{l}(l)}$ can be calculated using the log-likelihood of the EBNM model using the following Lemma 7.

**Lemma 7** *Suppose* $\overset{ˆ}{g},q$ *solves the EBNM problem with data* (x,s):

(A.42)
$$(\overset{ˆ}{g},q)=\text{EBNM}(x,s),$$

where $q≔\left(q_{1},\ldots ,q_{n}\right)$ are the estimated posterior distributions of the normal means parameters $\theta_{1},\ldots ,\theta_{n}$. Then

(A.43)
$$E_{q}\left(\text{log}\left(\underset{j}{\prod }\;\overset{ˆ}{g}\left(\theta_{j}\right)/\underset{j}{\prod }\;q_{j}\left(\theta_{j}\right)\right)\right)=l(\overset{ˆ}{g};x,s)+\frac{1}{2}\underset{j}{\sum }\;\text{log}\left(2\pi s_{j}^{2}\right)+\left(1/s_{j}^{2}\right)\left(x_{j}^{2}+E_{q}\left(\theta_{j}^{2}\right)-2x_{j}E_{q}\left(\theta_{j}\right)\right.$$

where $l(\overset{ˆ}{g};x,s)$ is the log of the likelihood for the normal means problem (A.27).

**Proof** We have from (A.28)

(A.44)
$$F^{\text{NM}}\left(q_{\theta },\hat{g}\right)=\int \;q_{\theta }\left(\theta \right)\text{log}\frac{p\left(x,\theta |\hat{g}\right)}{q_{\theta }\left(\theta \right)}d\theta$$

(A.45)
$$=\int \;q_{\theta }(\theta )\text{log}\frac{p(x|\theta )\overset{ˆ}{g}(\theta )}{q_{\theta }(\theta )}d\theta$$

(A.46)
$$=E_{q}\left(\text{log}\left(\prod_{j}\;\;\overset{ˆ}{g}\left(\theta_{j}\right)/\prod_{j}\;\;q_{j}\left(\theta_{j}\right)\right)\right)-\frac{1}{2}E_{q}\left[\sum_{j}\;\;\text{log}\left(2\pi s_{j}^{2}\right)+\left(1/s_{j}^{2}\right){\left(x_{j}-\theta_{j}\right)}^{2}\right]$$

And the result follows from noting that $F^{\text{NM}}(\overset{ˆ}{q},\overset{ˆ}{g})=l(\overset{ˆ}{g})$. ■

### A.5 Inference with Penalty Term

Conceivably, in some settings one might like to encourage solutions to the EBMF problem be sparser than the maximum-likelihood estimates for $g_{l},g_{f}$ would produce. This could be done by extending the EBMF model to introduce a penalty term on the distributions $g_{l},g_{f}$ so that the maximum likelihood estimates are replaced by maximizing a penalized likelihood. We are not advocating for this approach, but it is straightforward given existing machinery, and so we document it here for completeness.

Let $h_{l}\left(g_{l}\right)$ and $h_{f}\left(g_{f}\right)$ denote penalty terms on $g_{l}$ and $g_{f}$, so the penalized log-likelihood would be:

(A.47)
$$l\left(g_{l},g_{f},\tau \right)\;\;≔\text{log}\left[p\left(Y|g_{l},g_{f},\tau^{2}\right)\right]+h_{l}\left(g_{l}\right)+h_{f}\left(g_{f}\right)\;\;=F\left(q,g_{l},g_{f},\tau^{2}\right)+h_{l}\left(g_{l}\right)+h_{f}\left(g_{f}\right)+D_{KL}\left(q∥p\right)$$

where $F\left(q,g_{l},g_{f},\tau^{2}\right)$ and $D_{KL}(q∥p)$ are defined in (3.4) and (3.5). And the corresponding penalized variational objective is:

(A.48)
$$\text{max}F\left(q,g_{l},g_{f},\tau^{2}\right)+h_{l}\left(g_{l}\right)+h_{f}\left(g_{f}\right).$$

It is straightforward to modify the algorithms above to maximize this penalized objective: simply modify the EBNM solvers to solve a corresponding penalized normal means problem. That is, instead of estimating the prior g by maximum likelihood, the EBNM solver must now maximize the penalized log-likelihood:

(A.49)
$$\overset{ˆ}{g}=\text{arg}\underset{g\in 𝒢}{\text{max}}\;l_{\text{EBNM}}(g)+h(g),$$

where $l_{\text{EBNM}}$ denote the log-likelihood for the EBNM problem. (The computation of the posterior distributions given $\overset{ˆ}{g}$ is unchanged).

For example, the ashr software (Stephens, 2017) provides the option to include a penalty on g to encourage overestimation of the size of the point mass on zero. This penalty was introduced to ensure conservative behavior in False Discovery Rate applications of the normal means problem. It is unclear that such a penalty is desirable in the matrix factorization application. However, the above discussion shows that using this penalty (e.g. within the ebnm function used by the greedy or backfitting algorithms) can be thought of as solving a penalized version of the EBMF problem.

## Appendix B. Orthogonal Cross Validation

Cross-validation assessments involving “holding out” (hiding) data from methods. Here we introduce a novel approach to selecting the data to be held out, which we call Orthogonal Cross Validation (OCV). Although not the main focus of our paper, we believe that OCV is a novel and appealing approach to selecting hold-out data for factor models, e.g. when using CV to select an appropriate dimension K for dimension reduction methods, as in Owen and Wang (2016).

Generic k-fold CV involves randomly dividing the data matrix into k parts and then, for each part, training methods on the other k-1 parts before assessing error on that part, as in Algorithm 6.

The novel part of OCV is in how to choose the “hold-out” pattern. We randomly divide the columns and rows into k sets. and put these sets into k orthogonal parts, and then take all $Y_{ij}$ with the chosen column and row indices as “hold-out” $Y_{\left(i\right)}$.

To illustrate this scheme, we take 3-fold CV as an example. We randomly divide the columns into 3 sets and the rows into 3 sets as well. The data matrix Y is divided into 9 partition (by row and column permutation):

$$Y=\left(\begin{array}{lll} Y_{11} & Y_{12} & Y_{13}\\ Y_{21} & Y_{22} & Y_{23}\\ Y_{31} & Y_{32} & Y_{33} \end{array}\right)$$

Then $Y_{\left(1\right)}=\left\{Y_{11},Y_{22},Y_{33}\right\},Y_{\left(2\right)}=\left\{Y_{12},Y_{23},Y_{31}\right\}$ and $Y_{\left(3\right)}=\left\{Y_{13},Y_{21},Y_{32}\right\}$ are orthogonal to each other. Then the data matrix Y is marked as:

$$Y=\left(\begin{array}{lll} Y_{\left(1\right)} & Y_{\left(2\right)} & Y_{\left(3\right)}\\ Y_{\left(3\right)} & Y_{\left(1\right)} & Y_{\left(2\right)}\\ Y_{\left(2\right)} & Y_{\left(3\right)} & Y_{\left(1\right)} \end{array}\right)$$

In OCV, each fold $k,Y_{\left(k\right)}$ contains equally balanced part of data matrix and includes all the row and column indices. This ensures that all *i*’s and j’s are included into each $Y_{-(k)}$. In 3-fold OCV, we have:

(B.1)
$$Y=\left(\begin{array}{lll} Y_{11} & Y_{12} & Y_{13}\\ Y_{21} & Y_{22} & Y_{23}\\ Y_{31} & Y_{32} & Y_{33} \end{array}\right)=\left[\begin{array}{c} L^{\left(1\right)}\\ L^{\left(2\right)}\\ L^{\left(3\right)} \end{array}\right]\times \left[\begin{array}{lll} F^{\left(1\right)} & F^{\left(2\right)} & F^{\left(3\right)} \end{array}\right]+E$$

(B.2)
$$=\left(\begin{array}{lll} Y_{\left(1\right)} & Y_{\left(2\right)} & Y_{\left(3\right)}\\ Y_{\left(3\right)} & Y_{\left(1\right)} & Y_{\left(2\right)}\\ Y_{\left(2\right)} & Y_{\left(3\right)} & Y_{\left(1\right)} \end{array}\right)=\left[\begin{array}{lll} L^{\left(1\right)}F^{\left(1\right)} & L^{\left(1\right)}F^{\left(2\right)} & L^{\left(1\right)}F^{\left(3\right)}\\ L^{\left(2\right)}F^{\left(1\right)} & L^{\left(2\right)}F^{\left(2\right)} & L^{\left(2\right)}F^{\left(3\right)}\\ L^{\left(3\right)}F^{\left(1\right)} & L^{\left(3\right)}F^{\left(2\right)} & L^{\left(3\right)}F^{\left(3\right)} \end{array}\right]+E$$

where $Y_{\left(1\right)}=\left\{Y_{11},Y_{22},Y_{33}\right\},Y_{\left(2\right)}=\left\{Y_{12},Y_{23},Y_{31}\right\}$ and $Y_{\left(3\right)}=\left\{Y_{13},Y_{21},Y_{32}\right\}$. We can see for each “hold-out” part, $Y_{\left(k\right)},L^{\left(1\right)},L^{\left(2\right)},L^{\left(3\right)}$ and $F^{\left(1\right)},F^{\left(2\right)},F^{\left(3\right)}$ show up once and only once. In this sense the hold-out pattern is “balanced”.

## References

[R1] Argelaguet Ricard, Velten Britta, Arnol Damien, Dietrich Sascha, Zenz Thorsten, John C Marioni Florian Buettner, Huber Wolfgang, and Stegle Oliver. Multi-omics factor analysis—a framework for unsupervised integration of multi-omics data sets. Molecular systems biology, 14(6):e8124, 2018. 29925568 10.15252/msb.20178124 PMC6010767

[R2] Attias Hagai. Independent factor analysis. Neural Computation, 11(4):803–851, 1999. 10226184 10.1162/089976699300016458

[R3] Bai Jushan and Ng Serena. Large Dimensional Factor Analysis. Now Publishers Inc, 2008.

[R4] Bhattacharya Anirban and Dunson David B. Sparse Bayesian infinite factor models. Biometrika, pages 291–306, 2011. 23049129 10.1093/biomet/asr013 PMC3419391

[R5] Bishop Christopher M.. Variational principal components. In Ninth International Conference on Artificial Neural Networks (Conf. Publ. No. 470), volume 1, pages 509–514. IET, 1999.

[R6] Blei David M, Kucukelbir Alp, and McAuliffe Jon D. Variational inference: A review for statisticians. Journal of the American Statistical Association, 112(518):859–877, 2017.

[R7] Bouchard Guillaume, Naradowsky Jason, Riedel Sebastian, Rocktäschel Tim, and Vlachos Andreas. Matrix and tensor factorization methods for natural language processing. In Proceedings of the 53rd Annual Meeting of the Association for Computational Linguistics and the 7th International Joint Conference on Natural Language Processing: Tutorial Abstracts, pages 16–18, Beijing, China, July 2015. Association for Computational Linguistics. doi: 10.3115/v1/P15-5005. URL https://www.aclweb.org/anthology/P15-5005.

[R8] Breiman Leo and Friedman Jerome H.. Estimating optimal transformations for multiple regression and correlation. Journal of the American Statistical Association, 80(391):580–598, 1985. doi: 10.1080/01621459.1985.10478157. URL http://www.tandfonline.com/doi/abs/10.1080/01621459.1985.10478157.

[R9] Carvalho Carlos M., Chang Jeffrey, Lucas Joseph E., Nevins Joseph R., Wang Quanli, and West Mike. High-dimensional sparse factor modeling: Applications in gene expression genomics. Journal of the American Statistical Association, 103(484):1438–1456, 2008. ISSN 0162–1459. doi: 10.1198/016214508000000869. 21218139 PMC3017385

[R10] Clyde Merlise and George Edward I. Flexible empirical Bayes estimation for wavelets. Journal of the Royal Statistical Society Series B, 62(4):681–698, 2000. doi: 10.1111/1467-9868.00257.

[R11] Consortium GTEx The genotype-tissue expression (GTEx) pilot analysis: Multitissue gene regulation in humans. Science, 348(6235):648–660, 2015. 25954001 10.1126/science.1262110 PMC4547484

[R12] Ding Chris HQ, Li Tao, and Jordan Michael I. Convex and semi-nonnegative matrix factorizations. IEEE transactions on pattern analysis and machine intelligence, 32(1):45–55, 2008. 10.1109/TPAMI.2008.277 19926898

[R13] Eckart C and Young G. The approximation of one matrix by another of lower rank. Psychometrika, 1:211–218, 1936.

[R14] Engelhardt Barbara E and Stephens Matthew. Analysis of population structure: a unifying framework and novel methods based on sparse factor analysis. PLoS Genetics, 6(9): e1001117, sep 2010. 20862358 10.1371/journal.pgen.1001117 PMC2940725

[R15] Fithian William, Mazumder Rahul, Flexible low-rank statistical modeling with missing data and side information. Statistical Science, 33(2):238–260, 2018.

[R16] Ford J Kevin, MacCallum Robert C, and Tait Marianne. The application of exploratory factor analysis in applied psychology: A critical review and analysis. Personnel Psychology, 39(2):291–314, 1986.

[R17] Frühwirth-Schnatter Sylvia and Lopes Hedibert Freitas. Sparse Bayesian factor analysis when the number of factors is unknown. arXiv preprint arXiv:1804.04231, 2018.

[R18] Gao Chuan, Brown Christopher D, and Engelhardt Barbara E. A latent factor model with a mixture of sparse and dense factors to model gene gene expression data with confounding effects. arXiv:1310.4792v1, 2013.

[R19] Gao Chuan, McDowell Ian C., Zhao Shiwen, Brown Christopher D., and Engelhardt Barbara E.. Context specific and differential gene co-expression networks via Bayesian biclustering. PLoS Computational Biology, 12(7):1–39, 07 2016. doi: 10.1371/journal.pcbi.1004791. URL 10.1371/journal.pcbi.1004791. PMC4965098 27467526

[R20] Ghahramani Zoubin and Beal Matthew J. Variational inference for Bayesian mixtures of factor analysers. In Advances in neural information processing systems, pages 449–455, 2000.

[R21] Girolami Mark. A variational method for learning sparse and overcomplete representations. Neural Computation, 13(11):2517–2532, 2001. 11674849 10.1162/089976601753196003

[R22] Harper F Maxwell and Konstan Joseph A. The Movielens datasets: History and context. ACM Transactions on Interactive Intelligent Systems, 5(4):19, 2016.

[R23] Hastie Trevor, Mazumder Rahul, Lee Jason D, and Zadeh Reza. Matrix completion and low-rank SVD via fast alternating least squares. Journal of Machine Learning Research, 16(1):3367–3402, 2015. 31130828 PMC6530939

[R24] Hochreiter Sepp, Bodenhofer Ulrich, Heusel Martin, Mayr Andreas, Mitterecker Andreas, Kasim Adetayo, Khamiakova Tatsiana, Suzy Van Sanden Dan Lin, Talloen Willem, Bijnens Luc, Göhlmann Hinrich W. H., Shkedy Ziv, and Clevert Djork-Arné. FABIA: factor analysis for bicluster acquisition. Bioinformatics, 26(12):1520–1527, 04 2010. ISSN 1367–4803. doi: 10.1093/bioinformatics/btq227. URL 10.1093/bioinformatics/btq227. 20418340 PMC2881408

[R25] Hore Victoria, Ana Viñuela Alfonso Buil, Knight Julian, Mark I McCarthy Kerrin Small, and Marchini Jonathan. Tensor decomposition for multiple-tissue gene expression experiments. Nature Genetics, 48(9):1094–1100, 2016. 27479908 10.1038/ng.3624 PMC5010142

[R26] Jaakkola Tommi S. and Jordan Michael I.. Bayesian parameter estimation via variational methods. Statistics and Computing, 10:25–37, 2000.

[R27] Johnstone Iain M. and Silverman Bernard W.. Empirical Bayes selection of wavelet thresholds. The Annals of Statistics, 33(4):1700–1752, 2005a.

[R28] Johnstone Iain M and Silverman Bernard W. EBayesthresh: R and s-plus programs for empirical Bayes thresholding. J. Statist. Soft, 12:1–38, 2005b.

[R29] Johnstone Iain M, Silverman Bernard W, Needles and straw in haystacks: Empirical Bayes estimates of possibly sparse sequences. The Annals of Statistics, 32(4):1594–1649, 2004.

[R30] Jolliffe Ian T, Trendafilov Nickolay T, and Uddin Mudassir. A modified principal component technique based on the lasso. Journal of Computational and Graphical Statistics, 12(3): 531–547, 2003.

[R31] Josse Julie, Sardy Sylvain, and Wager Stefan. denoiseR: A package for low rank matrix estimation, 2018.

[R32] Kaufmann Sylvia and Schumacher Christian. Identifying relevant and irrelevant variables in sparse factor models. Journal of Applied Econometrics, 32(6):1123–1144, 2017.

[R33] Klami Arto. Polya-gamma augmentations for factor models. In Asian Conference on Machine Learning, pages 112–128, 2015.

[R34] Knowles David and Ghahramani Zoubin. Nonparametric Bayesian sparse factor. Annals of Applied Statistics, 5(2B):1534–1552, 2011. doi: 10.1214/10-AOAS435.

[R35] Koenker Roger and Mizera Ivan. Convex optimization, shape constraints, compound decisions, and empirical Bayes rules. Journal of the American Statistical Association, 109 (506):674–685, 2014a.

[R36] Koenker Roger and Mizera Ivan. Convex optimization in R. Journal of Statistical Software, 60(5):1–23, 2014b. doi: 10.18637/jss.v060.i05.

[R37] Kolda Tamara G and Bader Brett W. Tensor decompositions and applications. SIAM review, 51(3):455–500, 2009.

[R38] Lee DD and Seung HS. Learning the parts of objects by non-negative matrix factorization. Nature, 401(6755):788–791, 1999. doi: 10.1038/44565. 10548103

[R39] Lefrançais Emma, Ortiz-muñoz Guadalupe, Caudrillier Axelle, Mallavia Beñat, Liu Fengchun, Sayah David M, Thornton Emily E, Headley Mark B, David Tovo, Coughlin Shaun R, Krummel Matthew F, Leavitt Andrew D, Passegué Emmanuelle, and Looney Mark R. The lung is a site of platelet biogenesis and a reservoir for haematopoietic progenitors. Nature, 544(7648):105–109, 2017. doi: 10.1038/nature21706. 28329764 PMC5663284

[R40] Lim Yew Jin and Teh Yee Whye. Variational Bayesian approach to movie rating prediction. In Proceedings of KDD cup and workshop, volume 7, pages 15–21. Citeseer, 2007.

[R41] Mayrink Vinicius Diniz, Lucas Joseph Edward, Sparse latent factor models with interactions: Analysis of gene expression data. The Annals of Applied Statistics, 7(2): 799–822, 2013.

[R42] Mazumder Rahul, Hastie Trevor, and Tibshirani Robert. Spectral regularization algorithms for learning large incomplete matrices. Journal of Machine Learning Research, 11(Aug): 2287–2322, 2010. 21552465 PMC3087301

[R43] Nakajima Shinichi and Sugiyama Masashi. Theoretical analysis of Bayesian matrix factorization. Journal of Machine Learning Research, 12:2583–2648, 2011.

[R44] Nakajima Shinichi, Sugiyama Masashi, Babacan S Derin, and Tomioka Ryota. Global analytic solution of fully-observed variational bayesian matrix factorization. Journal of Machine Learning Research, 14(Jan):1–37, 2013.

[R45] Owen Art B and Wang Jingshu. Bi-cross-validation for factor analysis. Statistical Science, 31(1):119–139, 2016.

[R46] Pournara Iosifina and Wernisch Lorenz. Factor analysis for gene regulatory networks and transcription factor activity profiles. BMC bioinformatics, 8:61, 2007. ISSN 1471–2105. doi: 10.1186/1471-2105-8-61. PMC1821042 17319944

[R47] Raiko Tapani, Ilin Alexander, and Karhunen Juha. Principal component analysis for large scale problems with lots of missing values. In European Conference on Machine Learning, pages 691–698. Springer, 2007.

[R48] Ročková Veronika and George Edward I. Fast Bayesian factor analysis via automatic rotations to sparsity. Journal of the American Statistical Association, 111(516):1608–1622, 2016.

[R49] Rubin Donald B. Inference and missing data. Biometrika, 63(3):581–592, 1976.

[R50] Rubin Donald B and Thayer Dorothy T. EM algorithms for ML factor analysis. Psychometrika, 47(1):69–76, 1982.

[R51] Sabatti Chiara and James Gareth M. Bayesian sparse hidden components analysis for transcription regulation networks. Bioinformatics, 22(6):739–746, 2005. 16368767 10.1093/bioinformatics/btk017

[R52] Seeger Matthias and Bouchard Guillaume. Fast variational Bayesian inference for non-conjugate matrix factorization models. In Artificial Intelligence and Statistics, pages 1012–1018, 2012.

[R53] Srivastava Sanvesh, Engelhardt Barbara E, and Dunson David B. Expandable factor analysis. Biometrika, 104(3):649–663, 2017. 29430037 10.1093/biomet/asx030 PMC5793687

[R54] Stegle Oliver, Parts Leopold, Durbin Richard, and Winn John. A Bayesian framework to account for complex non-genetic factors in gene expression levels greatly increases power in eqtl studies. PLoS Computational Biology, 6(5):e1000770, 2010. 20463871 10.1371/journal.pcbi.1000770 PMC2865505

[R55] Stegle Oliver, Parts Leopold, Piipari Matias, Winn John, and Durbin Richard. Using probabilistic estimation of expression residuals (PEER) to obtain increased power and interpretability of gene expression analyses. Nature Protocols, 7(3):500–507, 2012. 22343431 10.1038/nprot.2011.457 PMC3398141

[R56] Stein-O’Brien Genevieve L, Arora Raman, Culhane Aedin C, Favorov Alexander V, Garmire Lana X, Greene Casey S, Goff Loyal A, Li Yifeng, Ngom Aloune, Ochs Michael F, Enter the matrix: factorization uncovers knowledge from omics. Trends in Genetics, 34 (10):790–805, 2018. 30143323 10.1016/j.tig.2018.07.003 PMC6309559

[R57] Stephens Matthew. False discovery rates: a new deal. Biostatistics, 18(2):275–294, Apr 2017. doi: 10.1093/biostatistics/kxw041. 27756721 PMC5379932

[R58] Thomas DC, Siemiatycki J, Dewar R, Robins J, Goldberg M, and Armstrong BG. The problem of multiple inference in studies designed to generate hypotheses. American Journal of Epidemiology, 122(6):1080–95, Dec 1985. 4061442 10.1093/oxfordjournals.aje.a114189

[R59] Tipping Michael E. Sparse Bayesian learning and the relevance vector machine. Journal of Machine Learning Research, 1(Jun):211–244, 2001.

[R60] Titsias Michalis K. and Lázaro-Gredilla Miguel. Spike and slab variational inference for multi-task and multiple kernel learning. In Shawe-Taylor J, Zemel RS, Bartlett PL, Pereira F, and Weinberger KQ, editors, Advances in Neural Information Processing Systems 24, pages 2339–2347. Curran Associates, Inc., 2011. URL http://papers.nips.cc/paper/4305-spike-and-slab-variational-inference-for-multi-task-and-multiple-kernel-learning.pdf.

[R61] Wang Wei. Applications of Adaptive Shrinkage in Multiple Statistical Problems. PhD thesis, The University of Chicago, 2017.

[R62] West Mike. Bayesian factor regression models in the “large p, small n” paradigm. Bayesian Statistics 7 - Proceedings of the Seventh Valencia International Meeting, pages 723–732, 2003. ISSN 08966273. doi: 10.1.1.18.3036.

[R63] Wipf David P. and Nagarajan Srikantan S.. A new view of automatic relevance determination. In Platt JC, Koller D, Singer Y, and Roweis ST, editors, Advances in Neural Information Processing Systems 20, pages 1625–1632. Curran Associates, Inc., 2008. URL http://papers.nips.cc/paper/3372-a-new-view-of-automatic-relevance-determination.pdf.

[R64] Witten Daniela M., Tibshirani Robert, and Hastie Trevor. A penalized matrix decomposition, with applications to sparse principal components and canonical correlation analysis. Biostatistics, 10(3):515–534, 2009. doi: 10.1093/biostatistics/kxp008. 19377034 PMC2697346

[R65] Xing Zhengrong, Carbonetto Peter, and Stephens Matthew. Smoothing via adaptive shrinkage (smash): denoising Poisson and heteroskedastic Gaussian signals. arXiv preprint arXiv:1605.07787, 2016.

[R66] Yang Dan, Ma Zongming, and Buja Andreas. A sparse singular value decomposition method for high-dimensional data. Journal of Computational and Graphical Statistics, 23(4):923–942, 2014.

[R67] Zhao Shiwen, Barbara E Engelhardt Sayan Mukherjee, and Dunson David B. Fast moment estimation for generalized latent Dirichlet models. Journal of the American Statistical Association, pages 1–13, 2018. 35875263 10.1080/01621459.2017.1341839 PMC9302535

[R68] Zou Hui, Hastie Trevor, and Tibshirani Robert. Sparse principal component analysis. Journal of Computational and Graphical Statistics, 15(2):265–286, 2006. ISSN 1061–8600. doi:10.1198/106186006X113430.

